# Cliques of Neurons Bound into Cavities Provide a Missing Link between Structure and Function

**DOI:** 10.3389/fncom.2017.00048

**Published:** 2017-06-12

**Authors:** Michael W. Reimann, Max Nolte, Martina Scolamiero, Katharine Turner, Rodrigo Perin, Giuseppe Chindemi, Paweł Dłotko, Ran Levi, Kathryn Hess, Henry Markram

**Affiliations:** ^1^Blue Brain Project, École Polytechnique Fédérale de LausanneGeneva, Switzerland; ^2^Laboratory for Topology and Neuroscience, Brain Mind Institute, École Polytechnique Fédérale de LausanneLausanne, Switzerland; ^3^Laboratory of Neural Microcircuitry, Brain Mind Institute, École Polytechnique Fédérale de LausanneLausanne, Switzerland; ^4^DataShape, INRIA SaclayPalaiseau, France; ^5^Institute of Mathematics, University of AberdeenAberdeen, United Kingdom

**Keywords:** connectomics, topology, directed networks, structure-function, correlations, Betti numbers

## Abstract

The lack of a formal link between neural network structure and its emergent function has hampered our understanding of how the brain processes information. We have now come closer to describing such a link by taking the direction of synaptic transmission into account, constructing graphs of a network that reflect the direction of information flow, and analyzing these directed graphs using algebraic topology. Applying this approach to a local network of neurons in the neocortex revealed a remarkably intricate and previously unseen topology of synaptic connectivity. The synaptic network contains an abundance of cliques of neurons bound into cavities that guide the emergence of correlated activity. In response to stimuli, correlated activity binds synaptically connected neurons into functional cliques and cavities that evolve in a stereotypical sequence toward peak complexity. We propose that the brain processes stimuli by forming increasingly complex functional cliques and cavities.

## 1. Introduction

How the structure of a network determines its function is not well understood. For neural networks specifically, we lack a unifying mathematical framework to unambiguously describe the emergent behavior of the network in terms of its underlying structure (Bassett and Sporns, 2017). While graph theory has been used to analyze network topology with some success (Bullmore and Sporns, 2009), current methods are usually constrained to analyzing how local connectivity influences local activity (Pajevic and Plenz, 2012; Chambers and MacLean, 2016) or global network dynamics (Hu et al., 2014), or how global network properties like connectivity and balance of excitatory and inhibitory neurons influence network dynamics (Renart et al., 2010; Rosenbaum et al., 2017). One such global network property is small-worldness. While it has been shown that small-worldness optimizes information exchange (Latora and Marchiori, 2001), and that adaptive rewiring during chaotic activity leads to small world networks (Gong and Leeuwen, 2004), the degree of small-worldness cannot describe most local network properties, such as the different roles of individual neurons.

Algebraic topology (Munkres, 1984) offers the unique advantage of providing methods to describe quantitatively both local network properties and the global network properties that emerge from local structure, thus unifying both levels. More recently, algebraic topology has been applied to functional networks between brain regions using fMRI (Petri et al., 2014) and between neurons using neural activity (Giusti et al., 2015), but the underlying synaptic connections (structural network) were unknown. Furthermore, all formal topological analyses have overlooked the direction of information flow, since they analyzed only undirected graphs.

We developed a mathematical framework to analyze both the structural and the functional topology of the network, integrating local and global descriptions, enabling us to establish a clear relationship between them. We represent a network as a directed graph, with neurons as the vertices and the synaptic connections directed from pre- to postsynaptic neurons as the edges, which can be analyzed using elementary tools from algebraic topology (Munkres, 1984). The structural graph contains all synaptic connections, while a functional graph is a sub-graph of the structural graph containing only those connections that are active within a specific time bin (i.e., in which a postsynaptic neuron fires within a short time of a presynaptic spike). The response to a stimulus can then be represented and studied as a time series of functional graphs.

Networks are often analyzed in terms of groups of nodes that are all-to-all connected, known as *cliques*. The number of neurons in a clique determines its size, or more formally, its *dimension*. In directed graphs it is natural to consider *directed cliques*, which are cliques containing a single source neuron and a single sink neuron and reflecting a specific *motif* of connectivity (Song et al., 2005; Perin et al., 2011), wherein the flow of information through a group of neurons has an unambiguous direction. The manner in which directed cliques bind together can be represented geometrically. When directed cliques bind appropriately by sharing neurons, and without forming a larger clique due to missing connections, they form *cavities* (“*holes*,” “*voids*”) in this geometric representation, with high-dimensional cavities forming when high-dimensional (large) cliques bind together. Directed cliques describe the flow of information in the network at the local level, while cavities provide a global measure of information flow in the whole network. Using these naturally arising structures, we established a direct relationship between the structural graph and the emergent flow of information in response to stimuli, as captured through time series of functional graphs.

**Figure 1:**
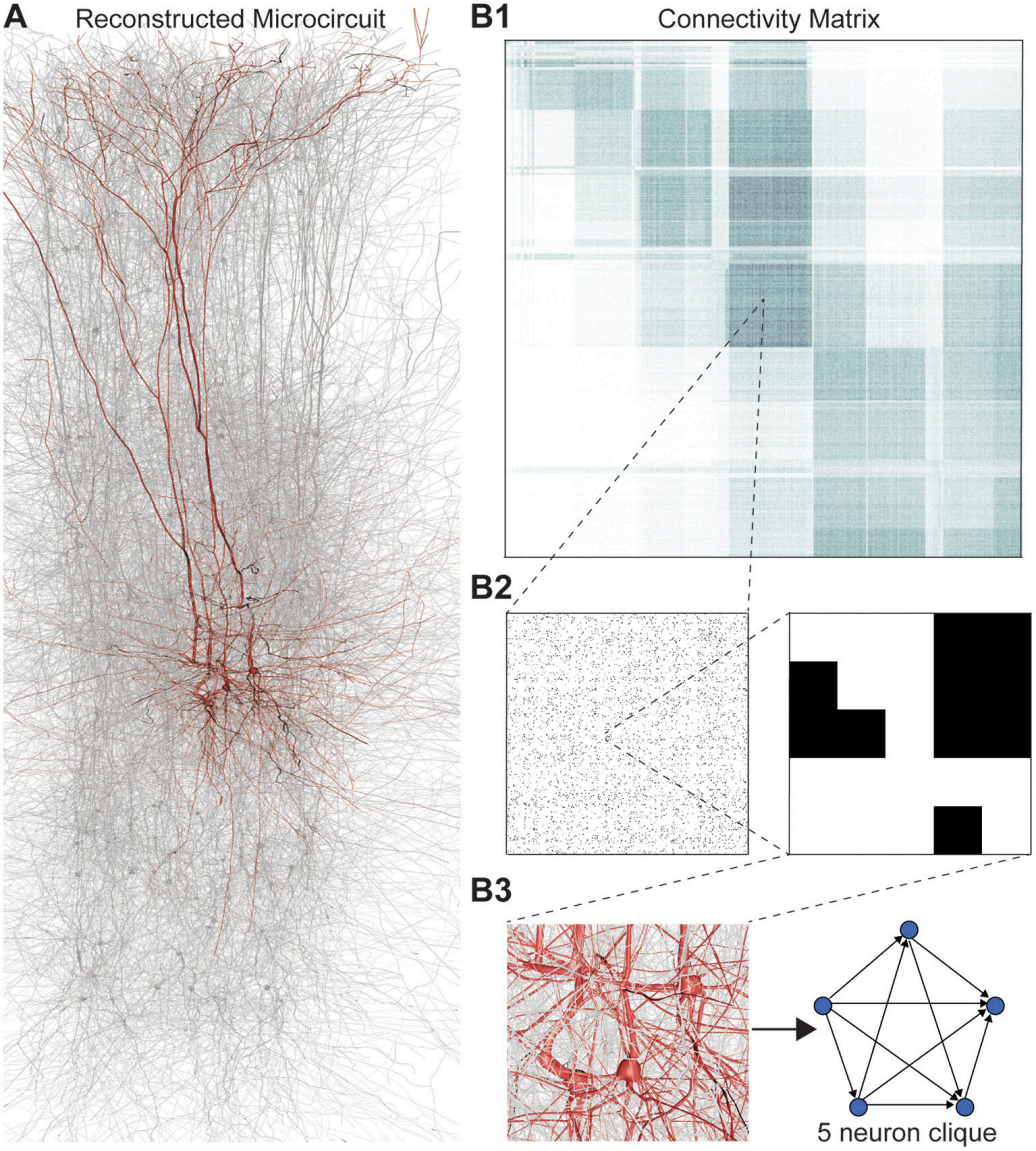
**(A)** Thin (10 μm) slice of *in silico* reconstructed tissue. Red: A clique formed by five pyramidal cells in layer 5. **(B1)** Full connection matrix of a reconstructed microcircuit with 31,146 neurons. Neurons are sorted by cortical layer and morphological type within each layer. Pre-/postsynaptic neurons along the vertical/horizontal axis. Each grayscale pixel indicates the connections between two groups of 62 neurons each, ranging from white (no connections) to black (≥8% connected pairs). **(B2)** Zoom into the connectivity between two groups of 434 neurons each in layer 5, i.e., 7 by 7 pixels in **(A)**, followed by a further zoom into the clique of 5 neurons shown in **(A)**. Black indicates presence, and white absence of a connection. **(B3)** Zoom into the somata of the clique in **(A)** and representation of their connectivity as a directed graph.

We applied this framework to digital reconstructions of rat neocortical microcircuitry that closely resemble the biological tissue in terms of the numbers, types, and densities of neurons and their synaptic connectivity (a “microconnectome” model for a cortical microcircuit, Figures 1A,B; see Markram et al., 2015; Reimann et al., 2015). Simulations of the reconstructed microcircuitry reproduce multiple emergent electrical behaviors found experimentally in the neocortex (Markram et al., 2015). The microcircuit, formed by ~8 million connections (edges) between ~31,000 neurons (vertices), was reconstructed from experimental data, guided by biological principles of organization, and iteratively refined until validated against a battery of independent anatomical and physiological data obtained from experiments. Multiple instantiations of the reconstruction provide a statistical and biological range of microcircuits for analysis.

We found a remarkably high number and variety of high-dimensional directed cliques and cavities, which had not been seen before in neural networks, either biological or artificial, and in far greater numbers than those found in various null models of directed networks. Topological metrics reflecting the number of directed cliques and cavities not only distinguished the reconstructions from all null models, they also revealed subtle differences between reconstructions based on biological datasets from different animals, suggesting that individual variations in biological detail of neocortical microcircuits are reflected in the repertoire of directed cliques and cavities. When we simulated microcircuit activity in response to sensory stimuli, we observed that pairwise correlations in neuronal activity increased with the number and dimension of the directed cliques to which a pair of neurons belongs, indicating that the hierarchical structure of the network shapes a hierarchy of correlated activity. In fact, we found a hierarchy of correlated activity between neurons even within a single directed clique. During activity, many more high-dimensional directed cliques formed than would be expected from the number of active connections, further suggesting that correlated activity tends to bind neurons into high-dimensional active cliques.

Following a spatio-temporal stimulus to the network, we found that during correlated activity, active cliques form increasingly high-dimensional cavities (i.e., cavities formed by increasingly larger cliques). Moreover, we discovered that while different spatio-temporal stimuli applied to the same circuit and the same stimulus applied to different circuits produced different activity patterns, they all exhibited the same general evolution, where functional relationships among increasingly higher-dimensional cliques form and then disintegrate.

## 2. Results

### 2.1. The case for directed simplices

Networks of neurons connected by electrical synapses (gap junctions) can be represented as undirected graphs, where information can flow in both directions. Networks with chemical synapses, which impose a single direction of synaptic communication from the pre- to the postsynaptic neuron (Figures 1B2,B3), are more accurately represented as directed graphs. Sub-sampling networks of neurons experimentally has revealed small motifs of synaptic connectivity, but not large cliques of neurons (Song et al., 2005; Perin et al., 2011). Knowing the complete directed network of neurons, as we do in the case of the reconstructed microcircuit, enables us to detect all cliques, directed, and otherwise (Figure 1).

**Figure 2:**
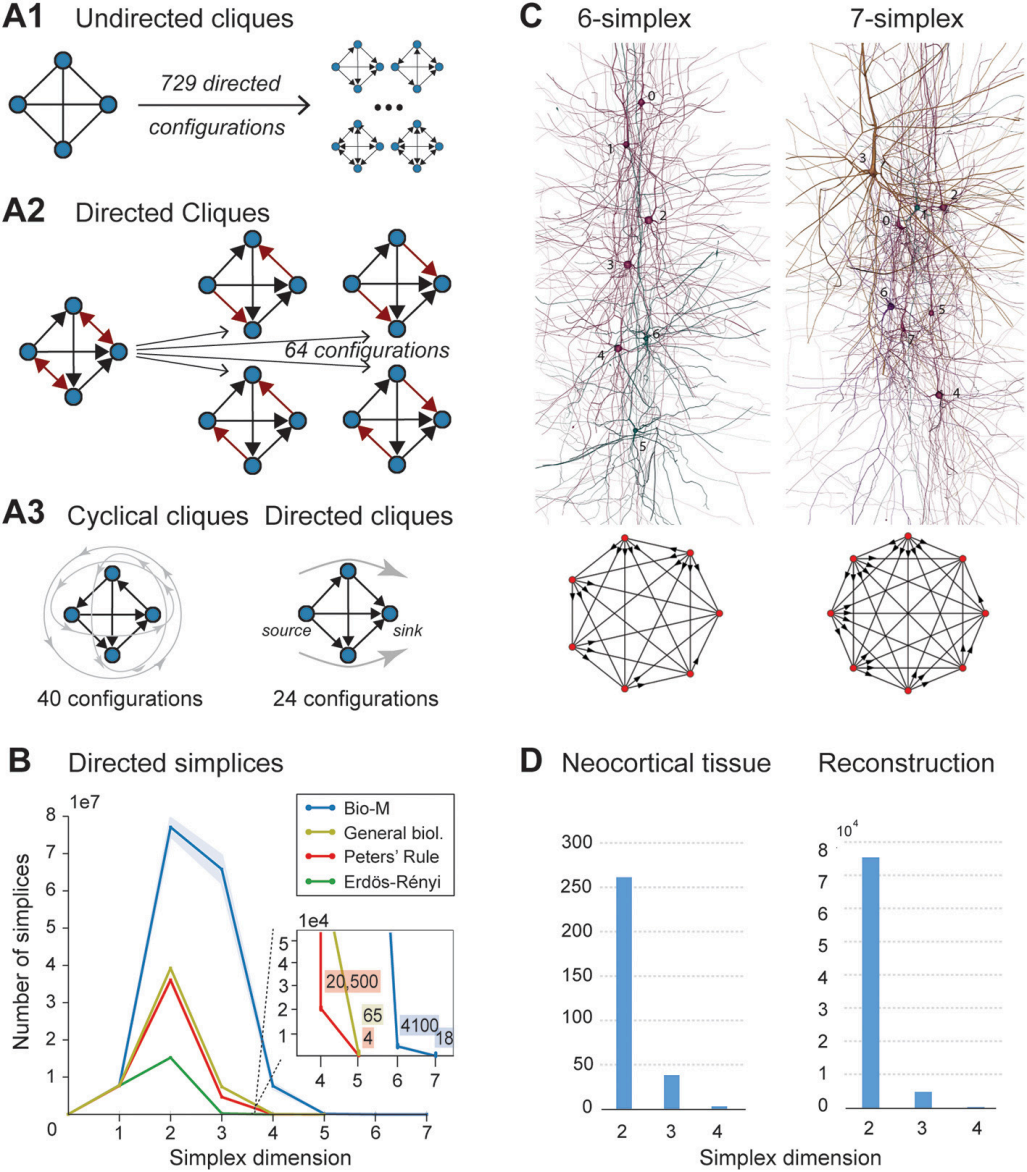
**(A1)** A 4-clique in the undirected connectivity graph has one of 729 *configurations* in the directed graph. **(A2)** Configurations containing bidirectional connections are resolved by considering all sub-graphs without bidirectional connections. **(A3)** Without bidirectional connections, 64 possible configurations remain, 24 of which are acyclic, with a clear sink-source structure (*directed simplices*, in this case of dimension 3). **(B)** Number of simplices in each dimension in the Bio-M reconstruction (shaded area: standard deviation of seven statistical instantiations) and in three types of random control networks. **(C)** Examples of neurons forming high-dimensional simplices in the reconstruction. Bottom: Their representation as directed graphs. **(D)** (Left) Number of directed simplices of various dimensions found in 55 *in vitro* patch-clamp experiments sampling groups of pyramidal cells in layer 5. (Right) Number of simplices of various dimensions found in 100,000 *in silico* experiments mimicking the patch-clamp procedure of **(B)**.

When the direction of connections is not taken into account, a great deal of information is lost. For example, in the undirected case, there is only one possible configuration for a clique of four fully connected neurons (Figure 2A1, left). However, in the directed case, there are 3^6^ = 729 possible configurations, as each of the six connections can be in one of three states (i → j, j ← i, or i ↔ j connection types; Figure 2A1 right).

A clique with reciprocal connections contains two or more cliques consisting only of uni-directional connections (Figure 2A2). When only uni-directional connections are considered, there are 2^6^ possible configurations of four fully connected neurons, which are of two types: those that contain cycles (40 configurations; Figure 2A3 left; Section 4.1.3) and those that do not (24 configurations; Figure 2A3 right). Directed cliques are exactly the acyclic cliques. The net *directionality* of information flow through any motif can be defined as the sum over all neurons of the squares of the differences between their in-degree and their out-degree (see Equation 2, Figure S1). Directed cliques have the highest net directionality among all cliques (Figure S1; Section 4.1.4). A clique that contains cycles always decomposes into directed cliques with the same number of neurons or fewer, at the very least any single connection between two neurons forms a 2-clique. A cyclical clique of three neurons therefore decomposes into three 2-cliques. Following the conventions in algebraic topology, we refer to directed cliques of *n* neurons as *directed simplices of dimension n-1* or *directed (n-1)-simplices* (which reflects their natural geometric representation as (*n*-1)-dimensional polyhedra) (see Figure S2; Section 4.1.3). Correspondingly, their sub-cliques are called *sub-simplices*.

### 2.2. An abundance of directed simplices

#### 2.2.1. Reconstructed neocortical microcircuitry

We analyzed 42 variants of the reconstructed microconnectome, grouped into six sets, each comprised of seven statistically varying instantiations (Markram et al., 2015; Section 4.3). The first five sets were based on specific heights of the six layers of the neocortex, cell densities, and distributions of different cell types experimentally measured in five different rats (Bio1-5), while the sixth represents the mean of these measurements (Bio-M). Individual instantiations within a set varied with the outcome of the stochastic portions of the reconstruction process. Surprisingly, we found that the reconstructions consistently contained directed simplices of dimensions up to 6 or 7, with as many as 80 million directed 3-simplices (Figure 2B; blue). This is the first indication of the existence of such a vast number of high-dimensional directed simplices in neocortical microcircuitry, or in any neural network.

#### 2.2.2. Control models

To compare these results with null models, we examined how the numbers of directed simplices in these reconstructions differed from those of artificial circuits and from circuits in which some of the biological rules of connectivity were omitted (see Section 4.4). For one control, we generated five Erdős-Rényi random graphs (ER) of equal size (~31,000 vertices) and the same average connection probability as the Bio-M circuit (~0.8%; ~8 million edges) (Figure 2B; dark green). For another, we constructed a circuit with the same 3D model neurons as the Bio-M circuit, but connected the neurons using a random connectivity rule [“Peters' Rule” (Peters and Feldman, 1976), PR; Figure 2B, red]. For the last control we connected the neurons in the Bio-M circuit according to the distance-dependent connection probabilities between the different morphological types of neurons. Since this control is similar to deriving connectivity from the average overlap of neuronal arbors (Shepherd et al., 2005), it retains the general biological (GB) features of connectivity between different types of neurons (Reimann et al., 2015), excluding only explicit pairwise connectivity between individual neurons, which is determined by the overlap of their *specific* arbors (Figure 2B, yellow). In all cases, the number of directed simplices of dimensions larger than 1 was far smaller than in the Bio-M circuit. In addition, the relative differences between the Bio-M and the null models increased markedly with dimension.

#### 2.2.3. *In vitro*

Simplices of high dimensions (such as those depicted in Figure 2C) have not yet been observed experimentally, as doing so would require simultaneous intracellular recording of large numbers of neurons. To obtain an indication of the presence of many high-dimensional directed simplices in the actual neocortical tissue, we performed multi-neuron patch-clamp experiments with up to 12 neurons at a time in *in vitro* slices of the neocortex of the same age and brain region as the digitally reconstructed tissue (Section 4.5.1). Although limited by the number of neurons we could simultaneously record from, we found a substantial number of directed simplices up to dimension 3, and even one 4-dimensional simplex, in just 55 multi-neuron recording experiments (Figure 2D, left). We then mimicked these experiments on the reconstructed microcircuit by repeating the same multi-neuron patch-clamp recordings *in silico* (Section 4.5.2) and found a similar shape of the distribution of 4-, 3-, and 2-simplices, though in lower frequencies than in the actual tissue (Figure 2D, right). These findings not only confirm that high-dimensional directed simplices are prevalent in the neocortical tissue, they also suggest that the degree of organization in the neocortex is even greater than that in the reconstruction, which is already highly significant (see Section 3).

#### 2.2.4. *C. elegans*

To test whether the presence of large numbers of high-dimensional directed simplices is a general phenomenon of neural networks rather than a specific phenomenon found in this part of the brain of this particular animal and at this particular age, we computed the numbers of directed simplices in the *C. elegans* connectome (Varshney et al., 2011) (Section 4.6). Again, we found many more high-dimensional simplices than expected from a random circuit with the same number of neurons (Figure S3).

#### 2.2.5. Simplicial architecture of neocortical microcircuitry

**Figure 3:**
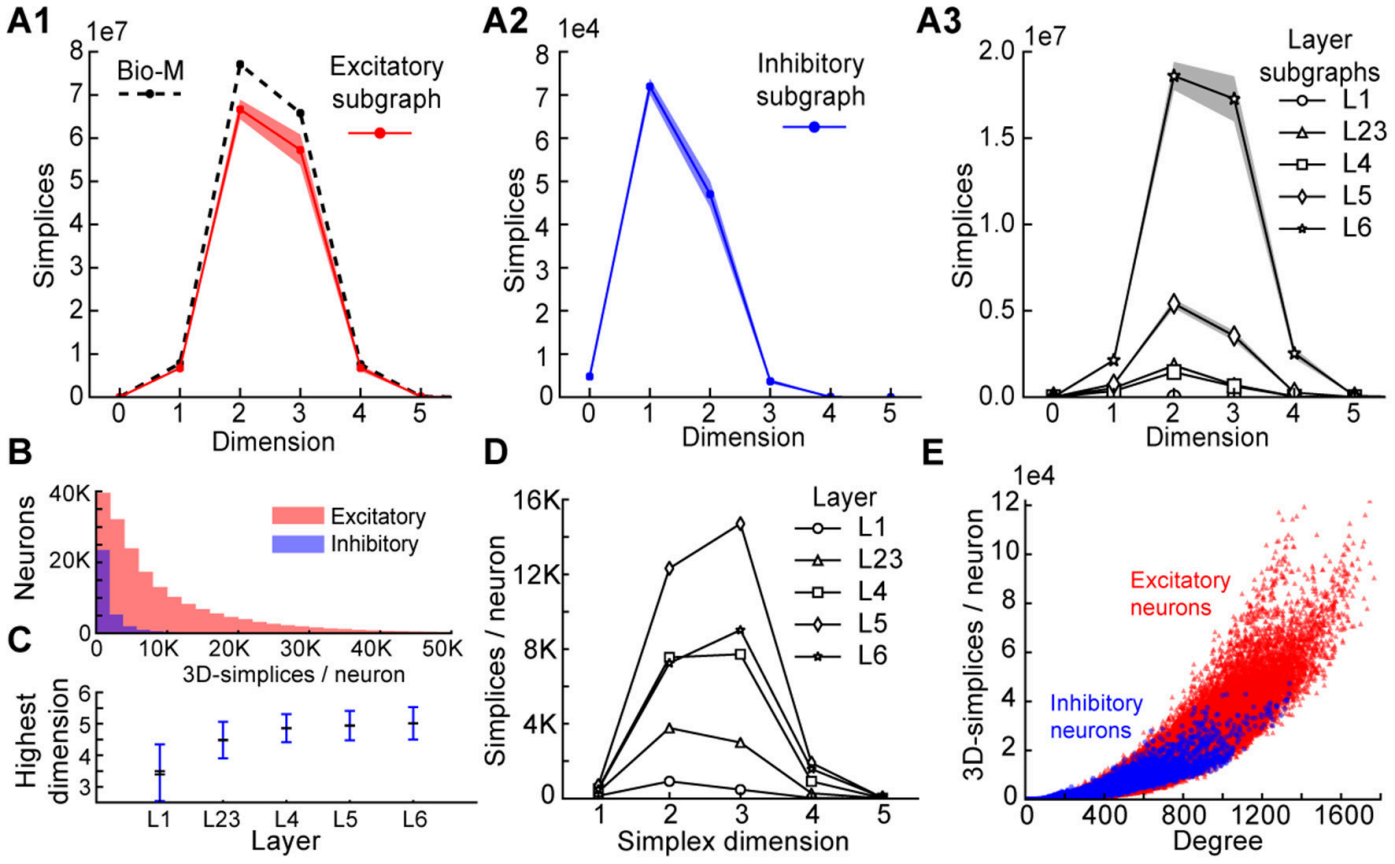
**(A1)** Number of simplices in each dimension in the excitatory subgraph (shaded area: standard deviation across seven instantiations). **(A2)** Same, for the inhibitory subgraph. **(A3)** Same, for the subgraphs of individual layers. **(B)** Distribution across seven instantiations of the Bio-M graph of the number of 3- simplices an excitatory (red) or inhibitory (blue) neuron belongs to (simplices/neuron). **(C)** Mean over neurons in individual layers of the highest dimension of a simplex that they belong to. **(D)** Simplices/neuron by layer and dimension. **(E)** Correlation of 3-simplices/neuron and degree in the graph for all neurons.

To understand the simplicial architecture of the microcircuit, we began by analyzing the sub-graphs formed only by excitatory neurons, only by inhibitory neurons, and only in individual layers by both excitatory and inhibitory neurons. Restricting to only excitatory neurons barely reduces the number of simplices in each dimension (Figure 3A1), while simplex counts in inhibitory sub-graphs are multiple orders of magnitude smaller (Figure 3A2), consistent with the fact that most neurons in the microcircuitry are excitatory. Analyzing the sub-graphs of the layers in isolation shows that layers 5 and 6, where most of the excitatory neurons reside (Markram et al., 2015), contain the most simplices and the largest number of high-dimensional simplices (Figure 3A3).

The large number of simplices relative to the number of neurons in the microcircuit implies that each neuron belongs to many directed simplices. Indeed, when we counted the number of simplices to which each neuron belongs across dimensions, we observed a long-tailed distribution such that a neuron belongs on average to thousands of simplices (Figure 3B). Both the mean maximal dimension and the number of simplices a neuron belongs to are highest in the deeper cortical layers (Figure 3C). Neurons in layer 5 belong to the largest number of simplices, many spanning multiple layers (Figure 3D), consistent with the abundance of neurons with the largest morphologies, which are connected to all layers. On the other hand, layer 6 has the largest number of simplices that are fully contained in the layer (Figure 3A3), consistent with the fact that layer 6 contains the most neurons. While the number of simplices that can form in the microcircuitry depends essentially on the number of neurons, the number of simplices to which a single neuron belongs depends fundamentally on its number of incoming and outgoing connections (its *degree*), which in turn depends on its morphological size (Figure 3E).

### 2.3. Topology organizes spike correlations

The presence of vast numbers of directed cliques across a range of dimensions in the neocortex, far more than in null models, demonstrates that connectivity between these neurons is highly organized into fundamental building blocks of increasing complexity. Since the structural topology of the neural network takes into account the direction of information flow, we hypothesized that emergent electrical activity of the microcircuitry mirrors its hierarchical structural organization. To test this hypothesis, we simulated the electrical activity of the microcircuit under *in vivo*-like conditions (Markram et al., 2015).

**Figure 4:**
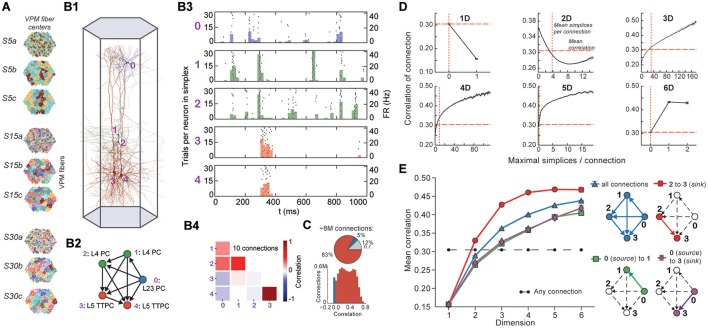
**(A)** Patterns of thalamic innervation in the reconstruction. Each circle represents the center of innervation of a thalamic fiber. Each color represents a unique thalamic spike train assigned to that fiber. **(B1)** Exemplary directed simplex in a microcircuit. **(B2)** Connectivity and morphological types of neurons in the exemplary simplex. **(B3)** Raster plot and PSTH (Δ*t* = 10 ms) of spiking response of neurons in **(B1,B2)** to stimulus S30b. **(B4)** Correlation coefficients of all pairs of PSTHs in **(B3)**. **(C)** Correlation coefficients of PSTHs for all stimuli and all connected pairs of neurons in a microcircuit (Δ*t* = 25 ms). **(D)** Mean correlation coefficients for connected pairs of neurons against the number of maximal simplices the edge between them belongs to, dimension by dimension. Means of fewer than 1,000 samples omitted. **(E)** Mean correlation coefficient of pairs of neurons, given their position within a simplex and its dimension.

Stimuli, configured as nine different spatio-temporal input patterns (Figure 4A), were injected into the reconstructed microcircuit through virtual thalamo-cortical fibers in which spike trains were induced using patterns recorded *in vivo* (Bale et al., 2015; Figure S4; Section 4.7). These stimuli differed primarily in the degree of synchronous input received by the neurons. As expected, the neurons in the microcircuit responded to the inputs with various spiking patterns (Figures 4B1,B2,B4). We then calculated for each connected pair of neurons the correlation of their spiking activity (Figure 4C) and found a broad distribution of correlation coefficients, with only ~12% of connections where either the pre- or postsynaptic neuron failed to respond during all stimuli.

To avoid redundant sampling when testing the relationship between simplex dimension and activity, we restricted our analysis to *maximal simplices*, i.e., directed simplices that are not part of any higher-dimensional simplex (Section 4.1.2). A connection can be part of many higher-dimensional maximal simplices, unless it is itself a maximal 1-simplex. Despite the restriction to maximal simplices, we retained all information about the structure of the microcircuit because the complete structure is fully determined by its list of maximal simplices (Section 4.1.2). Correlations were calculated from histograms of the average spiking response (peri-stimulus time histogram, PSTH; bin size, 25 ms) to five seconds of thalamo-cortical input over 30 repetitions of a given input pattern (Figure 4B3). We then calculated the normalized cross-covariance of the histograms for all connections (Figure 4C; Section 4.8) and compared it to the number of maximal simplices associated with each connection in each dimension (see Figure 4D).

The neurons forming maximal 1-simplices displayed a significantly lower spiking correlation than the mean (Figure 4D), an indication of the fragility and lack of integration of the connection into the network. The mean correlation initially decreased with the number of maximal 2-simplices a connection belongs to, and then increased slightly. We observed that the greater the number of maximal 2-simplices a connection belongs to, the less likely it is to belong to higher-dimensional maximal simplices, with the minimum correlation occurring when the connection belongs to no simplices of dimension higher than 3. In higher dimensions, the correlation increased with the number of maximal simplices to which a connection belongs. While very high mean correlation can be attained for connections belonging to many maximal 3- or 4-simplices, the mean correlation of connections belonging to just one maximal 5- or 6-simplex was already considerably greater than the mean. These findings reveal a strong relationship between the structure of the network and its emergent activity and specifically that spike correlations depend on the level of participation of connections in high-dimensional simplices.

To determine the full extent to which the topological structure could organize activity of neurons, we examined spike correlations between pairs of neurons within individual simplices. These correlations increased with simplex dimension (Figure 4E, blue), again demonstrating that the degree of organization in the activity increases with structural organization. Spike correlation between pairs of neurons is normally an ambiguous measurement of connection strength because it is influenced by the local structure, specifically by indirect connections and/or shared inputs (Palm et al., 1988; Brody, 1999). However, since in our case the local structure is known and described in terms of directed simplices, we could infer how the local structural organization influences spike correlations. We compared the impact of indirect connections and of shared inputs on correlated activity by calculating the average correlation of pairs of neurons at different positions in a simplex when ordered from source to sink (Figure 4E, right panel). The number of indirect connections is highest for the pair consisting of the first (source) and last (sink) neurons (Figure 4E, purple), while the number of shared inputs is highest for the last and second-to-last neurons (Figure 4E, red). The first (source) and second neurons (Figure 4E, green) serve as a control because they have the smallest numbers of both indirect connections and shared inputs in the simplex.

We found that correlations were significantly higher for the last two neurons in the simplex, suggesting that shared input generates more of the pairwise correlation in spiking than indirect connections in directed simplices (*p* < 8 · 10^−6^, all dimensions except 1D). Moreover, the spiking correlation of the source and sink neurons was similar to the correlation of the first and second neurons (Figure 4E, purple and green), further suggesting that spike correlations tend to increase as shared input increases. These results hold for a range of histogram time bin sizes (Figure S5). The specific positions of neurons in local structures such as directed simplices therefore shape the emergence of correlated activity in response to stimuli.

### 2.4. Cliques of neurons bound into cavities

**Figure 5:**
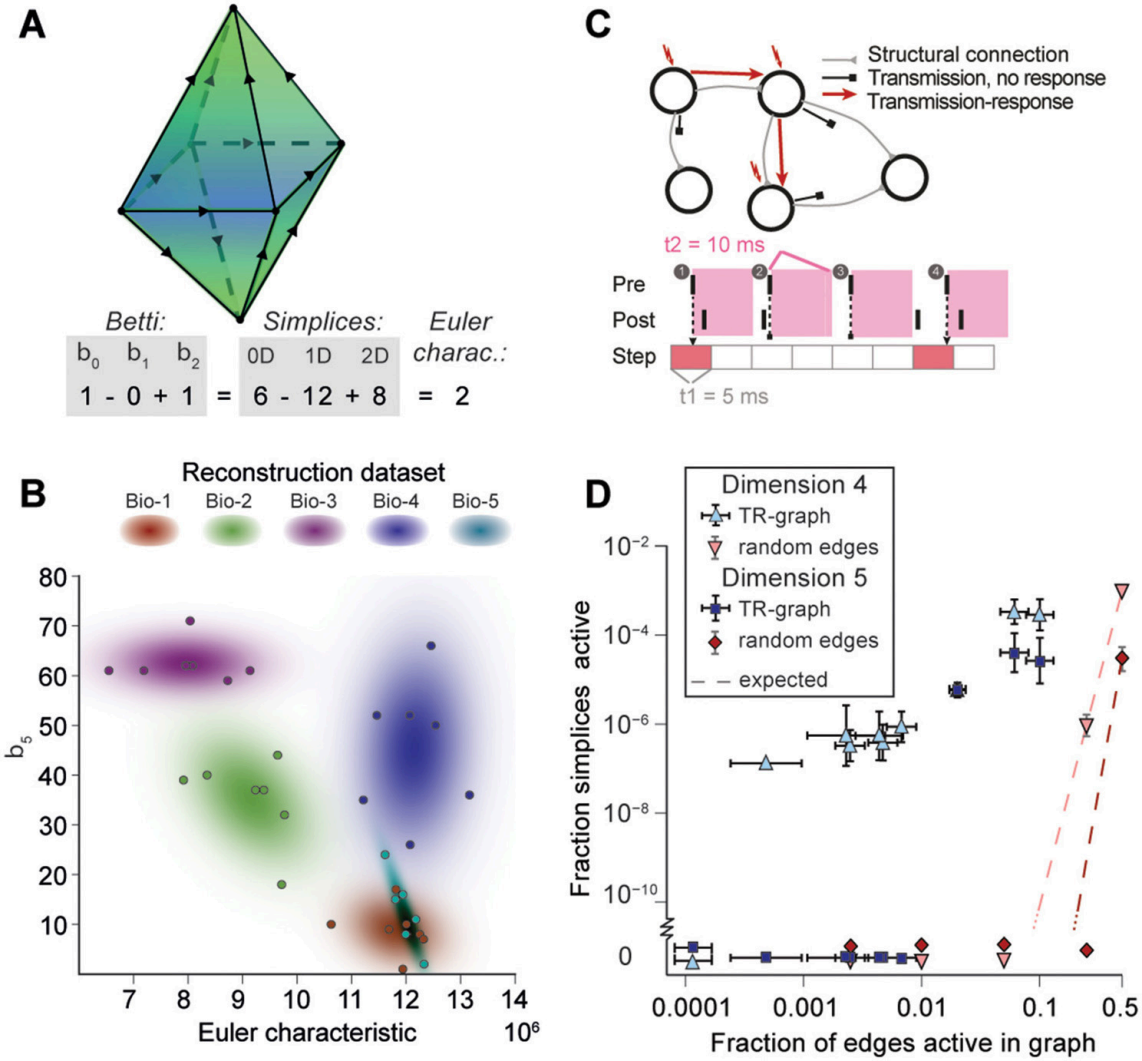
**(A)** Example of the calculation of the Euler characteristic of a directed flag complex as an alternating sum of Betti numbers or simplex counts. **(B)** Euler characteristic against the highest non-zero Betti number (β_5_) for seven instances of reconstructed microcircuits based on five different biological datasets (Bio 1-5). **(C)** Top: The transmission-response (TR) graph of the activity of a microcircuit is a subgraph of its structural connectivity containing all nodes, but only a subset of the edges (connections). Bottom: An edge is contained if its presynaptic neuron spikes in a defined time bin and its postsynaptic neurons spikes within 10 ms of the presynaptic spike. **(D)** Fraction of edges active against fraction of high-dimensional simplices active in TR graphs for various time bins of a simulation. Error bars indicate the standard deviation over 10 repetitions of the simulation. Blue triangles: 4-dimensional simplices, blue squares: 5-dimensional simplices. Red symbols and dashed lines indicate the results for choosing edges randomly from the structural graph and the number expected for random choice, respectively.

Simplices are the mathematical building blocks of the microcircuitry. To gain insight into how its global structure shapes activity, it is necessary to consider how simplices are bound together. This can be achieved by analyzing the *directed flag complex*, which is the set of all directed simplices together with the set of all sub-simplices for each simplex (Figure S6, Section 4.1.2). The directed flag complex is a complete representation of the graph, including in particular the cycles neglected when examining directed simplices in isolation. The relationship between any two directed simplices depends on how they share sub-simplices. Just as any simplex can be realized as a polyhedron, a directed flag complex can be realized as a geometric object, built out of these polyhedra. If two simplices share a sub-simplex, the corresponding polyhedra are glued together along a common face (Figure 5A). The “shape” (or, more precisely, the topology) of this geometric object fully describes the global structure of the network.

To analyze directed flag complexes we computed two descriptors, the *Euler characteristic* and *Betti numbers* (Section 4.1.5). The Euler characteristic of a flag complex is given by the alternating sum of the number of simplices in each dimension, from zero through the highest dimension (Figure 5A). The Betti numbers together provide an indication of the number of cavities (or more precisely, *homology classes*) fully enclosed by directed simplices in the geometric object realizing the directed flag complex, where the dimension of a cavity is determined by the dimension of the enclosing simplices. The *n*-th Betti number, denoted β_*n*_, indicates the number of n-dimensional cavities. For example, in Figure 5A, there is one 2-dimensional cavity (and therefore β_2_ = 1) enclosed by the eight triangles; if an edge were added between any two non-connected nodes, then the geometric object realizing the corresponding flag complex would be filled in with solid tetrahedra, and the cavity would disappear. In the flag complexes of the reconstructions, it was not possible to compute more than the zeroth and top nonzero Betti numbers, as lower dimensions were computationally too expensive (Section 4.2.2). We could easily compute all Betti numbers for the *C. elegans* connectome, however, as it has many fewer nodes and edges (Figure S3).

The Betti number computations showed that there are cavities of dimension 5 (cavities completely enclosed by 5-simplices/6-neuron directed cliques) in all seven instances of each of the reconstructions (Bio1-Bio5, Figure 5B; Bio-M not shown). In contrast, the ER- and PR-control models have no cavities of dimension higher than 3, and the GB-model has no cavities of dimension higher than 4, demonstrating that there are not only non-random building blocks in the reconstruction, but also non-random relationships among them. We found as well that the information encoded in β_5_ and the Euler characteristic together captures enough of the structure of the flag complex of a reconstruction to reveal subtle differences in their connectivity arising from the underlying biological data (Figure 5B, different colors).

### 2.5. Cliques and cavities in active sub-graphs

Thus far we have shown that the structural network guides the emergence of correlated activity. To determine whether this correlated activity is sufficiently organized to bind neurons together to form active cliques and to bind cliques together to form active cavities out of the structural graph, we represented the spiking activity during a simulation as a time series of sub-graphs for which we computed the corresponding directed flag complexes. Each sub-graph in this series comprises the same nodes (neurons) as the reconstruction, but only a subset of the edges (synaptic connections), which are considered *active*, i.e., the presynaptic neuron spikes in a time bin of size Δ*t*_1_ and the postsynaptic neuron spikes within a time Δ*t*_2_ after the presynaptic spike (Figure 5C and Figure S7, Section 4.9). By considering subsequent, non-overlapping time bins of constant size Δ*t*_1_, we obtain a time series of *transmission-response* (TR) graphs reflecting correlated activity in the microcircuitry. We converted the time series of TR graphs in response to the different patterns of thalamo-cortical inputs (see Figure 4A) into time series of directed flag complexes. We found significantly more simplices in the TR graphs (Δ*t*_1_ = 5 ms, Δ*t*_2_ = 10 ms) than would be expected based on the number of edges alone (Figure 5D), indicating that correlated activity becomes preferentially concentrated in directed simplices.

**Figure 6:**
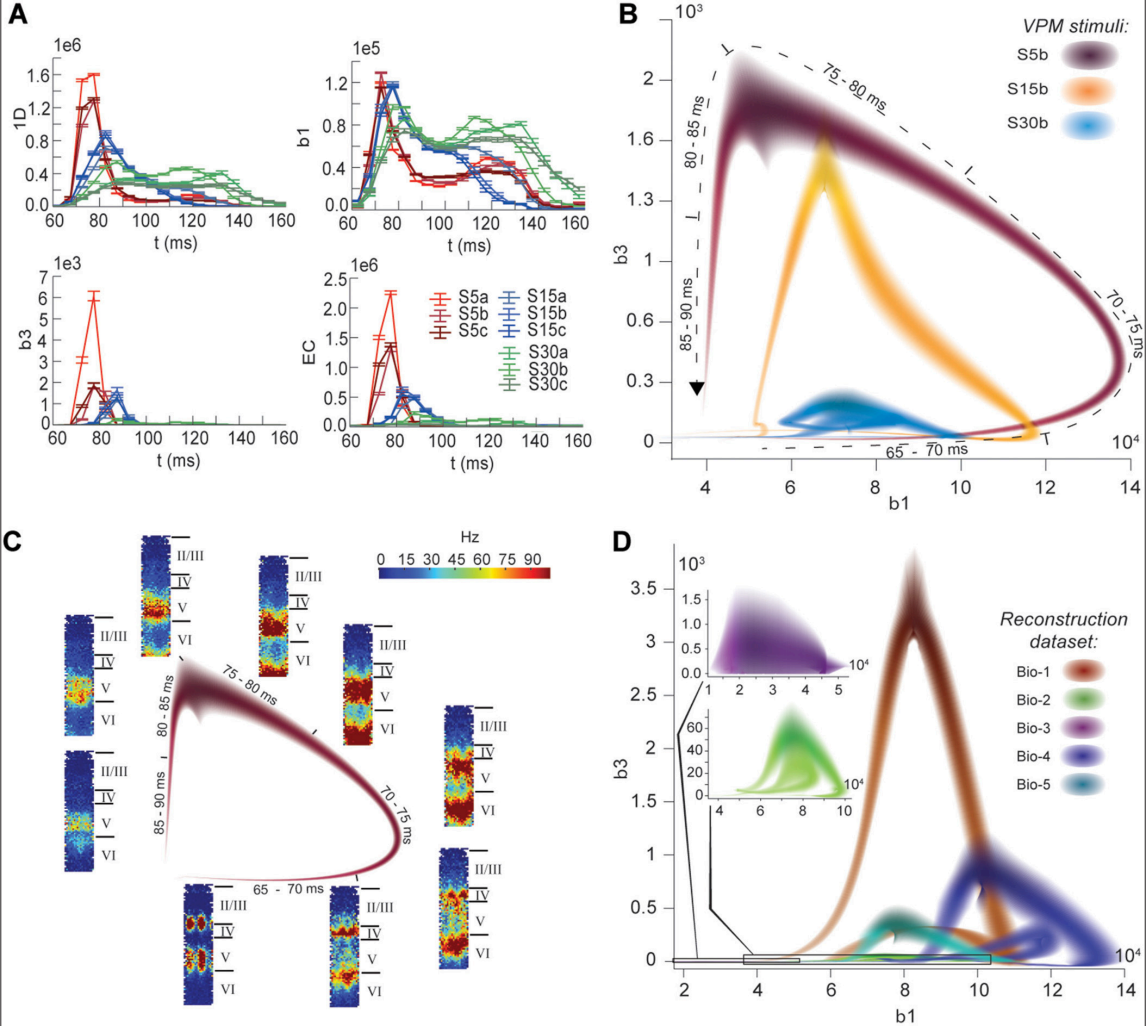
**(A)** Number of edges, β_1_, β_3_, and Euler characteristic of the time series of TR graphs in response to the stimulus patterns shown in Figure 4 (mean and SEM of 30 repetitions of each stimulus). **(B)** Trace of the time series of β_1_ against β_3_ for three of the stimuli. Shading of colors indicates Gaussian profiles at each time step with means and standard deviations interpolated from 30 repetitions of each stimulus. **(C)** Trace for one of the stimuli in B, along with the mean firing activity at different locations of the microcircuit during time steps of 2 ms. **(D)** Like **(B)**, but for TR graphs of Bio 1-5, in response to stimulus S15b.

The nine stimuli generated different spatio-temporal responses and different numbers of active edges (Figure 6A). The variation in Betti numbers and Euler characteristic over time indicates that neurons become bound into cliques and cavities by correlated activity (Figure 6A and Figure S8). When we plotted the number of cavities of dimension 1 (β_1_) against the number of those of dimension 3 (β_3_) (the highest dimension in which cavities consistently occur), the trajectory over the course of ~100 ms (Figure 6B) began ~50 ms after stimulus onset with the formation of a large number of 1-dimensional cavities, followed by the emergence of 2-dimensional (not shown) and 3-dimensional cavities. The decrease in β_1_ began while β_3_ was still increasing and continued until β_3_ reached its peak, indicating that higher-dimensional relationships between directed simplices continued to be formed by correlated activity as the lower dimensional relationships subside.

Different stimuli led to Betti number trajectories of different amplitudes, where higher degrees of synchrony in the thalamic input produced higher amplitudes. The trajectories all followed a similar progression of cavity formation toward a peak level of functional organization followed by relatively rapid disintegration. The center of the projection of each trajectory onto the β_1_-axis (its β_1_-center) was approximately the same. Together, these characteristics of the trajectories reveal a stereotypical evolution of cliques and cavities in response to stimuli. These observations are consistent with experimentally recorded *in vivo* responses to sensory stimuli in terms of onset delay, response duration, and the presence of distinct phases of the response (Luczak et al., 2015).

To determine the neurons involved in this robust evolution of functional organization, we recorded the mean levels of spiking activity at different spatial locations within the microcircuit for one exemplary stimulus (Figure 6C). The activity started at depths that correspond to the locations of the thalamo-cortical input (Meyer et al., 2010; Markram et al., 2015), increasing in layer 4 and at the top of layer 6, before propagating downwards, reaching the top of layer 5 and the center of layer 6 as β_1_ peaks, consistent with the finding that most directed simplices are in these layers. The transition from increasing β_1_ to increasing β_3_ coincided with the spread of the upper activity zone deeper into layer 5 and the top of layer 6, consistent with the presence of the highest dimensional directed simplices in these layers. The bottom activity zone also continued moving deeper, until it eventually subsided. As the top activity zone reached the bottom of layer 5, β_3_ attained its peak. The zones of activity at the peaks of β_1_ and β_3_ are highly complementary: zones active at the peak of β_1_ were generally inactive at the peak of β_3_ and vice versa. The activity zone then remained in layer 5 until the cavities collapsed.

Finally, we applied the same stimulus to the reconstructions based on variations in the underlying biological data (see Figure 5B, Bio-1 to 5) and found similar Betti number trajectories, indicating that the general sequence of cavity formation toward peak functional organization followed by disintegration is preserved across individuals. On the other hand, we observed markedly different amplitudes, indicating that biological variability leads to variation in the number of high-dimensional cavities formed by correlated activity (Figure 6D). We also found that, unlike the case of different stimuli applied to the same microcircuit (Figure 6B), trajectories arising from different biological variations have different β_1_-centers. In some cases, we observed reverberant trajectories that also followed a similar sequence of cavity formation, though smaller in amplitude. The general sequence of cavity formation and disintegration, however, appears to be stereotypic across stimuli and individuals.

## 3. Discussion

This study provides a simple, powerful, parameter-free, and unambiguous mathematical framework for relating the activity of a neural network to its underlying structure, both locally (in terms of simplices) and globally (in terms of cavities formed by these simplices). Using this framework revealed an intricate topology of synaptic connectivity containing an abundance of cliques of neurons and of cavities binding the cliques together. The study also provides novel insight into how correlated activity emerges in the network and how the network responds to stimuli.

Such a vast number and variety of directed cliques and cavities had not been observed before in any neural network. The numbers of high-dimensional cliques and cavities found in the reconstruction are also far higher than in null models, even in those closely resembling the biology-based reconstructed microcircuit, but with some of the biological constraints released. We verified the existence of high-dimensional directed simplices in actual neocortical tissue. We further found similar structures in a nervous system as phylogenetically different as that of the worm *C. elegans* (Varshney et al., 2011), suggesting that the presence of high-dimensional topological structures is a general phenomenon across nervous systems.

We showed that the spike correlation of a pair of neurons strongly increases with the number and dimension of the cliques they belong to and that it even depends on their specific position in a directed clique. In particular, spike correlation increases with proximity of the pair of neurons to the sink of a directed clique, as the degree of shared input increases. These observations indicate that the emergence of correlated activity mirrors the topological complexity of the network. While previous studies have found a similar link for motifs built from 2-dimensional simplices (Pajevic and Plenz, 2012; Chambers and MacLean, 2016), we generalize this to higher dimensions. The fact that each neuron belongs to many directed cliques of various dimensions explains *in vivo* observations that neurons can “flexibly join multiple ensembles” (Miller et al., 2014). Braids of directed simplices connected along their appropriate faces could possibly act as synfire chains (Abeles, 1982), with a superposition of chains (Bienenstock, 1995) supported by the high number of cliques each neuron belongs to.

Topological metrics reflecting relationships among the cliques revealed biological differences in the connectivity of reconstructed microcircuits. The same topological metrics applied to time-series of transmission-response sub-graphs revealed a sequence of cavity formation and disintegration in response to stimuli, consistent across different stimuli and individual microcircuits. The size of the trajectory was determined by the degree of synchronous input and the biological parameters of the microcircuit, while its location depended mainly on the biological parameters. Neuronal activity is therefore organized not only within and by directed cliques, but also by highly structured relationships between directed cliques, consistent with a recent hypothesis concerning the relationship between structure and function (Luczak et al., 2015).

The higher degree of topological complexity of the reconstruction compared to any of the null models was found to depend on the morphological detail of neurons, suggesting that the local statistics of branching of the dendrites and axons is a crucial factor in forming directed cliques and cavities, though the exact mechanism by which this occurs remains to be determined (but see Stepanyants and Chklovskii, 2005). The number of directed 2-, 3-, and 4-simplices found per 12-patch *in vitro* recording was higher than in the digital reconstruction, suggesting that the level of structural organization we found is a conservative estimate of the actual complexity. Since the reconstructions are stochastic instantiations at a specific age of the neocortex, they do not take into account rewiring driven by plasticity during development and learning. Rewiring is readily triggered by stimuli as well as spontaneous activity (Le Be and Markram, 2006), which leads to a higher degree of organization (Chklovskii et al., 2004; Holtmaat and Svoboda, 2009) that is likely to increase the number of cliques. The difference may also partly be due to incomplete axonal reconstructions that would lead to lower connectivity, but such an effect would be minor because the connection rate between the specific neurons recorded for this comparison is reasonably well constrained (Reimann et al., 2015).

The digital reconstruction does not take into account intracortical connections beyond the microcircuit. The increase in correlations between neurons with the number of cliques to which they belong should be unaffected when these connections are taken into account because the overall correlation between neurons saturates already for a microcircuit of the size considered in this study, as we have previously shown (Markram et al., 2015). However, the time course of responses to stimuli and hence the specific shape of trajectories may be affected by the neighboring tissue.

In conclusion, this study suggests that neocortical microcircuits process information through a stereotypical progression of clique and cavity formation and disintegration, consistent with a recent hypothesis of common strategies for information processing across the neocortex (Harris and Shepherd, 2015). We conjecture that a stimulus may be processed by binding neurons into cliques of increasingly higher dimension, as a specific class of cell assemblies, possibly to represent *features* of the stimulus (Hebb, 1949; Braitenberg, 1978), and by binding these cliques into cavities of increasing complexity, possibly to represent the *associations* between the features (Willshaw et al., 1969; Engel and Singer, 2001; Knoblauch et al., 2009).

## 4. Materials and methods

### 4.1. The topological toolbox

Specializing basic concepts of algebraic topology, we have formulated precise definitions of cliques (*simplices*) and cavities (as counted by *Betti numbers*) associated to directed networks. What follows is a short introduction to directed graphs, simplicial complexes associated to directed graphs, and homology, as well as to the notion of directionality in directed graphs used in this study. We define, among others, the following terms and concepts.

**Table:** 

**Term**	**Description**	**Section**
*Directed graph*	Network where each edge has a *source* and a *target*	4.1.1
*Simplex*	Clique of all-to-all connected nodes	4.1.2
*Directed simplex*	Simplex in a directed graph, with a *source* and a *sink*	4.1.3
*Source (of simplex)*	The node that is only a source of edges in a directed simplex	4.1.3
*Sink (of simplex)*	The node that is only a target of edges in a directed simplex	4.1.3
*Face (of simplex)*	Obtained by leaving out one or more nodes of a simplex	4.1.2
*Simplicial complex*	A collection of simplices “glued” together along common faces	4.1.2
*Maximal simplex*	Not a face of any larger simplex	4.1.2
*Directionality*	Formalized, intuitive measure of directionality in a graph	4.1.4
*Betti numbers*	Description of a graph in terms of the number of *cavities*	4.1.5.1
*Euler characteristic*	Alternating sum of number of simplices	4.1.5.2

#### 4.1.1. Directed graphs

A *directed graph* G consists of a pair of finite sets (*V, E*) and a function τ = (τ_1_, τ_2_): *E* → *V* × *V*. The elements of the set *V* are the *vertices* of G, the elements of *E* are the *edges* of G, and the function τ associates with each edge an ordered pair of vertices. The *direction* of an edge *e* with τ(*e*) = (*v*_1_, *v*_2_) is taken to be from τ_1_(*e*) = *v*_1_, the *source vertex*, to τ_2_(*v*) = *v*_2_, the *target vertex*. The function τ is required to satisfy the following two conditions.

1. There are no (self-) loops in the graph (i.e., for each *e* ∈ *E*, if τ(*e*) = (*v*_1_, *v*_2_), then *v*_1_ ≠ *v*_2_).
2. For any pair of vertices (*v*_1_, *v*_2_), there is at most one edge directed from *v*_1_ to *v*_2_ (i.e., the function τ is injective).

Notice that a directed graph may contain pairs of vertices that are reciprocally connected, i.e., there may exist edges *e, e*′ ∈ *E* such that τ(*e*) = (*v*_1_, *v*_2_) and $\tau \left(e\prime \right)=\left(v_{2},v_{1}\right)$ (Figure S6A1ii).

A vertex v∈G is said to be a *sink* if there exists no *e* ∈ *E* such that *v* = τ_1_(*e*), but there is at least one edge *e*′ ∈ *E* such that $\tau_{2}\left(e\prime \right)=v$. Similarly *v* is said to be a *source* is if there exists no *e* ∈ *E* such that *v* = τ_2_(*e*), but there is at least one *e*′ ∈ *E* such that $\tau_{1}\left(e\prime \right)=v$ (Figures S6A1i,iii). A *path* in a directed graph G consists of a sequence of edges (*e*_1_, …, *e*_*n*_) such that for all 1 ≤ *k* < *n*, the target of *e*_*k*_ is the source of *e*_*k*+1_, i.e., τ_2_(*e*_*k*_) = τ_1_(*e*_*k*+1_) (Figure S6A1iii). The *length* of the path (*e*_1_, …, *e*_*n*_) is *n*. If, in addition, the target of *e*_*n*_ is the source of *e*_1_, i.e., τ_2_(*e*_*n*_) = τ_1_(*e*_1_), then (*e*_1_, …, *e*_*n*_) is an *oriented cycle*. A graph that contains no oriented cycles is said to be *acyclic* (Figure S6A1i).

A directed graph is said to be *fully connected* if for every pair of distinct vertices, there exists an edge from one to the other, in at least one direction.

#### 4.1.2. Simplices, simplicial complexes, and flag complexes

An *abstract directed simplicial complex* is a collection S of finite, *ordered* sets with the property that if σ∈S, then every subset τ of σ, with the natural ordering inherited from σ, is also a member of S. A *subcomplex* of an abstract directed simplicial complex is a sub-collection S′⊆S that is itself an abstract directed simplicial complex. Abstract directed simplicial complexes are a variation on the more common ordinary *abstract simplicial complexes*, where the sets forming the collection S are not assumed to be ordered. To be able to study directed graphs, we use this slightly more subtle concept. Henceforth, we always refer to abstract directed simplicial complexes as *simplicial complexes*.

The elements σ of a simplicial complex S are called its *simplices*. We define the *dimension* of σ (denoted dim(σ)) to be the cardinality of the set σ minus one. If σ is a simplex of dimension *n*, then we refer to σ as an *n-simplex* of S. The set of all *n*-simplices of S is denoted $S_{n}$. A simplex τ is said to be a *face* of σ if τ is a subset of σ of a strictly smaller cardinality. The *front face* of an *n*-simplex σ = (*v*_0_, …, *v*_*n*_) is a face τ = (*v*_0_, …, *v*_*m*_) for some *m* < *n*. Similarly, the *back face* of σ is a face $\tau \prime =\left(v_{i},\ldots ,v_{n}\right)$ for some 0 < *i* < *n*. If $\sigma =\left(v_{0},\ldots ,v_{n}\right)\in S_{n}$ then, for each 0 ≤ *i* ≤ *n*, the *i*^*th*^ *face* of σ is the (*n*−1)-simplex σ^*i*^ obtained from σ by removing the vertex *v*_*n* − *i*_. A simplex that is not a face of any other simplex is said to be *maximal*. The set of all maximal simplices of a simplicial complex determines the entire simplicial complex, since every simplex is either maximal itself or a face of a maximal simplex.

A simplicial complex gives rise to a topological space by *geometric realization*. A 0-simplex is realized by a single point, a 1-simplex by a line segment, a 2-simplex by a (filled in) triangle, and so on for higher dimensions. (see Munkres, 1984, Section 1). To form the geometric realization of the simplicial complex, one then glues the geometrically realized simplices together along common faces. The intersection of two simplices in S, neither of which is a face of the other, is a proper subset, and hence a face, of both of them. In the geometric realization this means that the geometric simplices that realize the abstract simplices intersect on common faces, and hence give rise to a well-defined geometric object.

If S is a simplicial complex, then the union $S^{\left(n\right)}=S_{n}\cup \cdots \cup S_{0}$, called the *n-skeleton* of S, is a subcomplex of S. We say that S is *n-dimensional* if $S=S^{\left(n\right)}$, and *n* is minimal with this property. If S is *n*-dimensional, and *k* ≤ *n*, then the collection $S_{k}\cup \ldots \cup S_{n}$ is not a subcomplex of S because it is not closed under taking subsets. However, if one adds to that collection all the faces of all simplices in $S_{k}\cup \ldots \cup S_{n}$, one obtains a subcomplex of S called the *k-coskeleton* of S, which we will denote by $S_{\left(k\right)}$. Coskeleta are important for computing homology (see Section 4.2.2).

#### 4.1.3. Simplicial complexes of directed graphs

Directed graphs give rise to directed simplicial complexes in a natural way. The directed simplicial complex associated to a directed graph G is called the *directed flag complex* of G (Figure S6A2). This concept is a variation on the more common construction of a *flag complex* associated with an undirected graph (Aharoni et al., 2005). If G=(V,E,τ) is a directed graph, then the directed flag complex associated to G is the abstract directed simplicial complex S=S(G), with $S_{0}=V$ and whose *directed n-simplices* $S_{n}$ for *n* ≥ 1 are (*n* + 1)-tuples (*v*_0_, …, *v*_*n*_), of vertices such that for each 0 ≤ *i* < *j* ≤ *n*, there is an edge in G directed from *v*_*i*_ to *v*_*j*_. The vertex *v*_0_ is called the *source* of the simplex (*v*_0_, …, *v*_*n*_), as there is an edge directed from *v*_0_ to *v*_*i*_ for all 0 < *i* ≤ *n*. Conversely, the vertex *v*_*n*_ is called the *sink* of the simplex (*v*_0_, …, *v*_*n*_), as there is an edge directed from *v*_*i*_ to *v*_*n*_ for all 0 ≤ *i* < *n*.

Notice that because of the assumptions on τ, an *n*-simplex in S is characterized by the (ordered) sequence (*v*_0_, …, *v*_*n*_), but not by the underlying set of vertices. For instance (*v*_1_, *v*_2_, *v*_3_) and (*v*_2_, *v*_1_, *v*_3_) are distinct 2-simplices with the same set of vertices.

#### 4.1.4. Directionality of directed graphs

We give a mathematical definition of the notion of directionality in directed graphs, and prove that directed simplices are fully connected directed graphs with maximal directionality. Let G=(V,E,τ) be a directed graph. For each vertex v∈G, define the *signed degree* of *v* to be

(1) $$\begin{array}{l} \text{sd}\left(v\right)=\text{Indeg}\left(v\right)-\text{Outdeg}\left(v\right). \end{array}$$

Note that for any finite graph G, $\underset{v\in G}{\sum }\text{sd}\left(v\right)=0$. We define the *directionality* of G, denoted Dr(G), to be the sum over all vertices of the square of their signed degrees (Figure S1),

(2) $$\begin{array}{l} Dr\left(G\right)=\underset{v\in V}{\sum }sd{\left(v\right)}^{2}. \end{array}$$

Let $G_{n}$ denote a directed *n*-simplex, i.e., a fully connected directed graph on *n* + 1 vertices such that every complete subgraph has a unique source and a unique sink. Note that a directed *n*-simplex has no reciprocal connections. If G is any directed graph on *n* + 1 vertices, then $Dr\left(G\right)\leq Dr\left(G_{n}\right)$. If additionally G is a fully connected directed graph without reciprocal connections, then equality holds if and only if G is isomorphic to $G_{n}$ as a directed graph. A full proof of these statements is given in the Supplementary Methods.

#### 4.1.5. Homology

*Betti numbers* and *Euler characteristic* are numerical quantities associated to simplicial complexes that arise from an important and very useful algebraic object one can associate with any simplicial complex, called *homology*. Homology serves to measure the “topological complexity” of simplicial complexes, leading us to refer to Betti numbers and Euler characteristic as *topological metrics*. In this study we use only *mod 2 simplicial homology*, computationally the simplest variant of homology, which is why it is very commonly used in applications (Bauer et al., 2017). What follows is an elementary description of homology and its basic properties.

##### 4.1.5.1. Betti numbers

Let 𝔽_2_ denote the field of two elements. Let S be a simplicial complex. Define the *chain complex* $C_{*}\left(S,{𝔽}_{2}\right)$ to be the sequence ${\left\{C_{n}=C_{n}\left(S,{𝔽}_{2}\right)\right\}}_{n\geq 0}$, such that *C*_*n*_ is the 𝔽_2_-vector space whose basis elements are the *n*-simplices $\sigma \in S_{n}$, for each *n* ≥ 0. In other words, the elements of *C*_*n*_ are formal sums of *n*-simplices in S.

For each *n* ≥ 1, there is a linear transformation called a *differential*

(3) $$\begin{array}{l} \partial_{n}:C_{n}\to C_{n-1} \end{array}$$

specified by $\partial_{n}\left(\sigma \right)=\sigma^{0}+\sigma^{1}+\cdots +\sigma^{n}$ for every *n*-simplex σ, where σ^*i*^ is the *i*-th face of σ, as defined above. Having defined ∂_*n*_ on the basis, one then extends it linearly to the entire vector space *C*_*n*_. The *n*-th Betti number $\beta_{n}\left(S\right)$ of a simplicial complex S is the 𝔽_2_-vector space dimension of its *n*-th mod 2 *homology group*, which is defined by

(4) $$\begin{array}{l} H_{n}\left(S,{𝔽}_{2}\right)=\mathrm{Ker}\left(\partial_{n}\right)/Im\left(\partial_{n+1}\right) \end{array}$$

for *n* ≥ 1 and

(5) $$\begin{array}{l} H_{0}\left(S,{𝔽}_{2}\right)=C_{0}/Im\left(\partial_{1}\right). \end{array}$$

For all *n* ≥ 1, there is an inclusion of vector subspaces Im(∂_*n* + 1_) ⊆ Ker(∂_*n*_) ⊆ *C*_*n*_, and thus the definition of homology makes sense.

Computing the Betti numbers of a simplicial complex is conceptually very easy. Let $\left|S_{n}\right|$ denote the number of *n*-simplices in the simplicial complex S. If one encodes the differential ∂_*n*_ as a ($\left|S_{n-1}|\times |S_{n}\right|$)-matrix *D*_*n*_ with entries in 𝔽_2_, then one can easily compute its *nullity*, null(∂_*n*_), and its *rank*, rk(∂_*n*_), which are the 𝔽_2_-dimensions of the null-space and the column space of *D*_*n*_, respectively. The *Betti numbers* of S are then a sequence of natural numbers defined by

6 $$\begin{array}{l} \beta_{0}\left(S\right)=\dim_{{𝔽}_{2}}\left(C_{0}\right)-\mathrm{rk}\left(\partial_{1}\right),\text{ and}\\ \beta_{n}\left(S\right)=\mathrm{null}\left(\partial_{n}\right)-\mathrm{rk}\left(\partial_{n+1}\right). \end{array}$$

Since Im(∂_*n* + 1_) ⊆ Ker(∂_*n*_) for all *n* ≥ 1, the Betti numbers are always non-negative. The *n*-th Betti number β_*n*_ gives an indication of the number of “*n*-dimensional cavities” in the geometric realization of S.

##### 4.1.5.2. Euler characteristic

If S is a simplicial complex, and $\left|S_{n}\right|$ denotes the cardinality of the set of *n*-simplices in S, then the Euler characteristic of S is defined to be

(7) $$\begin{array}{l} \chi \left(S\right)=\underset{n\geq 0}{\sum }{\left(-1\right)}^{n}|S_{n}|. \end{array}$$

There is a well-known, close relationship between Euler characterstic and Betti numbers (Munkres, 1984, Theorem 22.2), which is expressed as follows. If ${\left\{\beta_{n}\left(S\right)\right\}}_{n\geq 0}$ is the sequence of Betti numbers for S, then

(8) $$\begin{array}{l} \chi \left(S\right)=\underset{n\geq 0}{\sum }{\left(-1\right)}^{n}\beta_{n}\left(S\right). \end{array}$$

### 4.2. Computation of simplices and homology

#### 4.2.1. Generating directed flag complexes with hasse diagrams

To obtain the simplices, Betti numbers and Euler characteristic of a directed graph, we first generate the directed flag complex associated to the graph. Our algorithm encodes a directed graph and its flag complex as a *Hasse diagram*. The Hasse diagram then gives immediate access to all simplices and simplex counts. The algorithm to generate the Hasse diagrams is fully described in the Supplementary Methods Section 2.2, and the C++ implementation of the code is publicly available at http://neurotop.gforge.inria.fr/.

#### 4.2.2. Homology computations

Betti numbers and Euler characteristic are computed from the directed flag complexes. All homology computations carried out for this paper were made with 𝔽_2_ coefficients, using the boundary matrix reduced by an algorithm from the PHAT library (Bauer et al., 2017).

The complexity of computing the *n*-th Betti numbers scales with the number of simplices in dimensions *n* − 1, *n*, and *n* + 1. In particular, it requires the computation of rank and nullity of matrices with shapes (*n* − 1) × *n* and *n* × (*n* + 1). Due to the millions of simplices in dimensions 2 and 3 in the reconstructed microcircuits (see Results), the calculation of Betti numbers above 0 or below 5 was computationally not viable, while the computation of the 5th Betti number was possible using the 5-coskeleton for each of the complexes. Nevertheless, our Euler characteristic computations imply that at least one of β_2_ or β_4_ must be nonzero, and it is highly likely the β_*k*_ is nonzero for all *k* ≤ 5.

### 4.3. Model of neocortical microcircuitry

Analyses of connectivity and simulations of electrical activity are based on a previously published model of neocortical microcircuitry and related methods (Markram et al., 2015). We analyzed microcircuits that were reconstructed with layer height and cell density data from five different animals (Bio-1-5), with seven microcircuits per animal forming a mesocircuit (35 microcircuits in total). In addition, we analyzed microcircuits that were reconstructed using average data (Bio-M, seven microcircuits). Simulations were run on one microcircuit each of Bio-1-5 and Bio-M. Each microcircuit contains ~31,000 neurons and ~8 million connections. Data about the microcircuit and the neuron models used in the simulations, as well as the connection matrices, are available on https://bbp.epfl.ch/nmc-portal/ (Ramaswamy et al., 2015).

### 4.4. Control networks

Additional control models of connectivity were constructed by removing different biological constraints on connectivity. We created three types of random matrices of sizes and connection probabilities identical to the connectivity matrices of the reconstructed microcircuits.

#### 4.4.1. ER-model (random-independent graph)

An empty square connection matrix of the same size as the connection matrix of the reconstruction was instantiated and then randomly selected off-diagonal entries were activated. Specifically, entries were randomly selected with equal probabilities until the same number of entries as in the reconstruction were active. The directed graph corresponding to such a matrix is the directed analog of an Erdős-Rényi random graph (Erdos and Rényi, 1960).

#### 4.4.2. PR-model (morphology-only, “peters' rule”)

A square connection matrix was generated based on the existence of spatial appositions between neurons in the reconstruction, i.e., instances where the axon of one neuron is within 1 μ*m* of a dendrite of the other neuron. Appositions were then randomly removed from the matrix with equal probabilities until the same number of connections as in the reconstruction remained.

#### 4.4.3. GB-model (shuffled, preserving distance dependance)

The connection matrix of a reconstructed microcircuit was split into 55^2^ submatrices based on the morphological types of pre- and postsynaptic neurons. Each submatrix was then randomized by shuffling its connections as follows. Connections in a sub-matrix were first grouped into bins according to the distance between the somata of their pre- and postsynaptic cells. Next, for each connection a new postsynaptic target was randomly selected from the same distance bin. We selected a distance bin size of 75μ*m*, which was the largest bin size that preserved the distribution of soma-distances of connected pairs of neurons in all sub-matrices (no statistically significant difference; *p* > 0.05, KL-test).

### 4.5. Patch clamp experiments

#### 4.5.1. *In vitro*

Connectivity between layer 5 thick-tufted pyramidal cells was analyzed using multiple somatic whole-cell recordings (6–12 cells simultaneously) on 300 μ*m* slices of primary somatosensory cortex of 14- to 16-day-old rats. Monosynaptic, direct excitatory connections were identified by stimulation of a presynaptic cell with a 20-70 Hz train of 5-15 strong and brief current pulses (1–2 nA, 2–4 ms). Experiments were carried out according to the Swiss national and institutional guidelines. Further details are explained in the Supplementary Methods.

#### 4.5.2. *In silico*

In order to obtain *in silico* cell groups comparable to their patched *in vitro* counterparts, we designed a cell selection procedure approximating several of the experimental constraints of the *in vitro* patch-clamp setup used in this study and explained above. In brief, layer 5 thick-tufted pyramidal cells were selected from a volume with dimensions of 200 × 200 × 20 μ*m*. The size of the volume was chosen to match the field of view usually available in the *in vitro* patch-clamp setup and to account for the tendency to patch nearby cells, which increases the probability of finding connected cells. The total number of cells was then reduced by randomly discarding a fraction of them, approximating the limited number of patching pipettes available *in vitro* (12) and the failure rate of the patching. This filtering step was optimized to match the *in silico* and *in vitro* cluster size distributions.

### 4.6. *C. elegans* connectome

We analyzed part of the *C. elegans* connectome (Varshney et al., 2011), consisting of 6,393 directed chemical synapses, obtained from www.wormatlas.org/neuronalwiring.html.

### 4.7. Simulation of electrical activity

We performed simulations of neuronal electrical activity during stimulation with spatio-temporal patterns of thalamic input at the *in vivo*-like state (as in Markram et al., 2015), in the central microcircuit of Bio-M. Additionally, we repeated the same simulations in the central microcircuits of the Bio-1-5 reconstructions. We ran simulations using nine different organizations of thalamic input spike trains (see below).

#### 4.7.1. Thalamic stimulation

We used spike trains of 42 VPM neurons extracted from extracellular recordings of the response to texture-induced whisker motion in anesthetized rats, with up to nine cells in the same barreloid recorded simultaneously (Bale et al., 2015). Each reconstructed microcircuit is innervated by 310 virtual thalamo-cortical fibers (Markram et al., 2015). To generate sets of stimuli with different degrees of synchronous input, we assigned to each fiber one of 5 (SS5), 15 (SS15), or 30 (SS30) spike trains, recorded from distinct VPM neurons. In addition, we used k-means clustering to form clusters of fibers of size 1 (SSa), 5 (SSb), and 10 (SSc) (scikit-learn, sklearn.cluster.KMeans, Pedregosa et al., 2011) that were assigned the same spike train. This leads to different spatial arrangements of the identical thalamic inputs, and therefore to different degrees of synchronous input to individual neurons in the microcircuit.

### 4.8. Spike train correlations

We constructed postsynaptic time histograms (PSTHs) for each neuron for each stimulus, using the mean response to 30 trials of 5 s of thalamic stimulation (with bin size of 25 ms; for additional control, bin sizes of 10, 50, 100, 250, and 500 ms were also used). We then computed the normalized covariance matrix of the PSTHs of all neurons

(9) $$\begin{array}{l} R_{ij}=\frac{C_{ij}}{\sqrt{C_{ii}C_{jj}}}, \end{array}$$

where *C*_*ij*_ is the covariance of the PSTHs of neurons *i* and *j*. PSTHs of simulations with different thalamic stimuli were concatenated for each neuron to yield an average correlation coefficient for all stimuli. In total, correlations are based on the response of all neurons during 30 trials of nine stimuli for 5 s of activity (22.5 min).

### 4.9. Transmission-response matrices

The temporal sequence of transmission-response matrices associated to a simulation of neuronal activity of duration *T* is defined as

(10) $$\begin{array}{l} TR\left(\Delta t_{1},\Delta t_{2}\right)\;:={\left\{A\left(n\right)=A\left(n,\Delta t_{1},\Delta t_{2}\right)\right\}}_{n=1}^{N}, \end{array}$$

where the *n*-th matrix, *A*(*n*), is a binary matrix describing spiking activity in the time interval [*n*·Δ*t*_1_, (*n* + 1)·Δ*t*_1_ + Δ*t*_2_], and where *N* = *T*/Δ*t*_1_. The (*j, k*)-coefficient of *A*(*n*) corresponding to the *n*-th time bin is 1 if and only if the following three conditions are satisfied, where $s_{i}^{j}$ denotes the time of the *i*-th spike of neuron *j*.

1. The (*j, k*)-coefficient of the structural matrix is 1, i.e., there is a structural connection from neuron *j* to neuron *k*, so that they form a pre-post synaptic pair.
2. There is some *i* such that $n\Delta t_{1}\leq s_{i}^{j}<\left(n+1\right)\Delta t_{1}$, i.e., neuron *j* spikes in the *n*-th time bin.
3. There is some *l* such that $0<s_{l}^{k}-s_{i}^{j}<\Delta t_{2}$, i.e., neuron *k* spikes after neuron *j*, within a Δ*t*_2_ interval.

In other words, a non-zero entry in a transmission-response matrix denotes a presynaptic spike, closely followed by a postsynaptic spike, maximizing the possibility of a causal relationship between the spikes. Based on firing data from spontaneous activity in the reconstructed microcircuit, we optimized Δ*t*_*i*_ such that the resulting transmission-response matrices best reflect the actual sucessful transmission of signals between the neurons in the microcircuit (see Supplementary Methods). Unless noted otherwise, Δ*t*_1_ = 5 and Δ*t*_2_ = 10 ms were used throughout the study.

### 4.10. Data analysis and statistical tests

Analysis of the model and simulations was performed on a Linux computing-cluster using Python 2.7, including the numpy and scipy libraries (Jones et al., 2001), and custom Python scripts. We calculated *p*-values using Welch's *t*-test (scipy.stats), unless noted otherwise.

## Author contributions

HM and RL developed and initially conceived the study over 10 years of discussions. HM, RL, and KH conceived and directed the final study. KH and RL directed the applicability of concepts in algebraic topology to neuroscience. HM directed the relevance of algebraic topology in neuroscience. The Blue Brain Project team reconstructed the microcircuit and developed the capability to simulate the activity. MN performed the simulations. MN, MR, and PD generated the directed flag complexes from the connection matrices for analysis. KH and RL developed the theory for directed cliques and directed simplicial complexes. MR and RL developed the definition of directionality within motifs and directed cliques. MR developed the definition for transmission response matrices. PD developed the code to isolate simplices and directed simplices and performed initial computations. MS performed topological and statistical analyses on the flag complexes and on the *C. elegans* connectome. KT helped with initial statistical analysis of network responses to stimuli. MR and MN analyzed the simulation data, mapped it onto the topological data and generated the figures. RP performed the patch-clamp experiments. GC and MR performed the corresponding *in silico* experiments. HM, KH, RL, MR, and MN wrote the paper.

### Conflict of interest statement

The authors declare that the research was conducted in the absence of any commercial or financial relationships that could be construed as a potential conflict of interest.
