# Supplementary material for: Cliques of Neurons Bound into Cavities Provide a Missing Link between Structure and Function
Source: Front Comput Neurosci. 2017 Jun 12;11:48. doi: 10.3389/fncom.2017.00048 (PMC5467434; doi:10.3389/fncom.2017.00048)
Supplement: Supplementary file 1 [file DataSheet1.pdf]

Supplementary Material:

Cliques of Neurons Bound into Cavities Provide a Missing Link between Structure and Function

Michael W. Reimann, Max Nolte, Martina Scolamiero, Katharine Turner, Rodrigo Perin, Giuseppe Chindemi, Paweł Dłotko, Ran Levi, Kathryn Hess * and Henry Markram *

*Correspondence:
henry.markram@epfl.ch
kathryn.hess@epfl.ch

Supplementary Material contains:

- Supplementary Figures (S1-8)
- Supplementary Methods (2.1-4)

1 SUPPLEMENTARY FIGURES

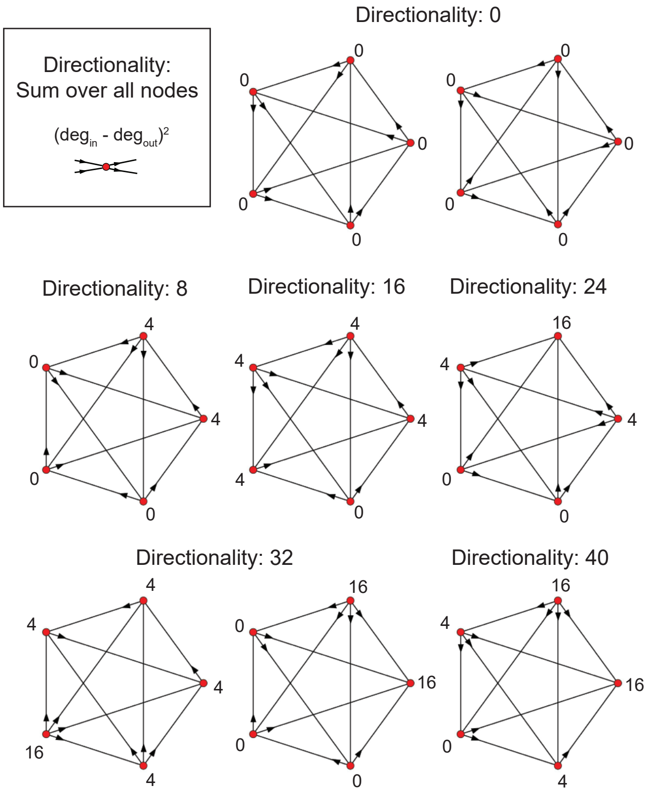

Figure S1: Examples of 5-cliques with various degrees of directionality, as well as the square of the difference of the in-degree and out-degree of all their nodes.

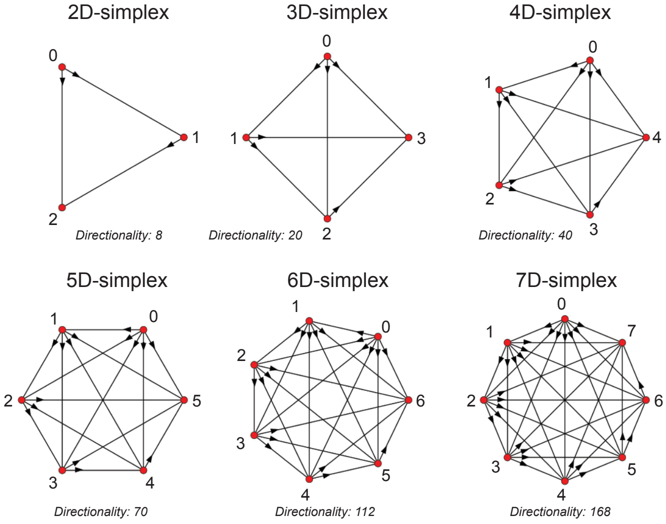

Figure S2: Directed simplices of dimensions 2-7, directionality as in Figure S1

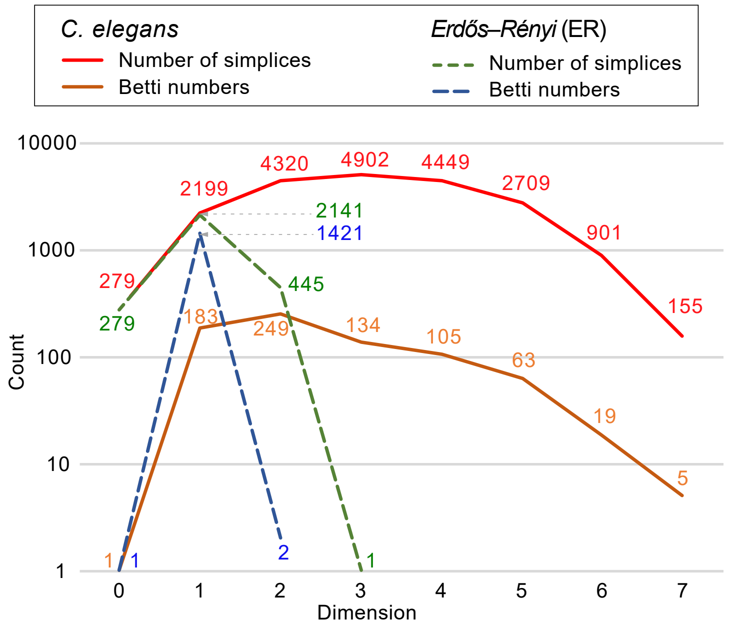

Figure S3: The number of simplices and Betti numbers for the C. elegans connectome, and for an ER-graph with the same number of nodes and the same connection probability.

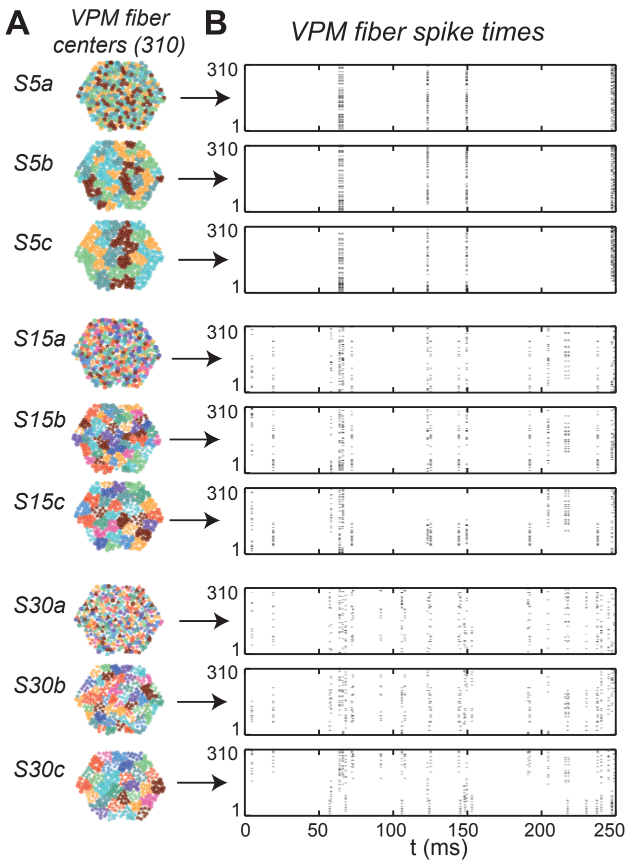

Figure S4: A: Same as Figure 4A. Each symbol represents the center of innervation of one of 310 thalamic fibers. B: Spike trains assigned to each thalamic fiber, during first 250 ms of each stimulus.

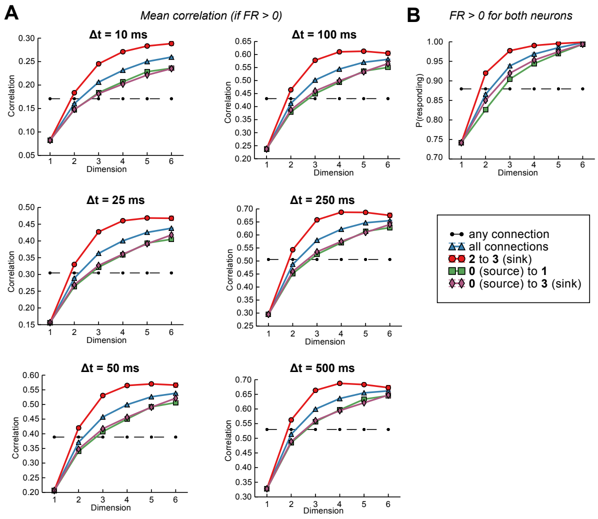

Figure S5: A: Same as Figure 4E, but for different PSTH time bin sizes. B: Probability of both neurons in a connection firing at least once during all stimuli.

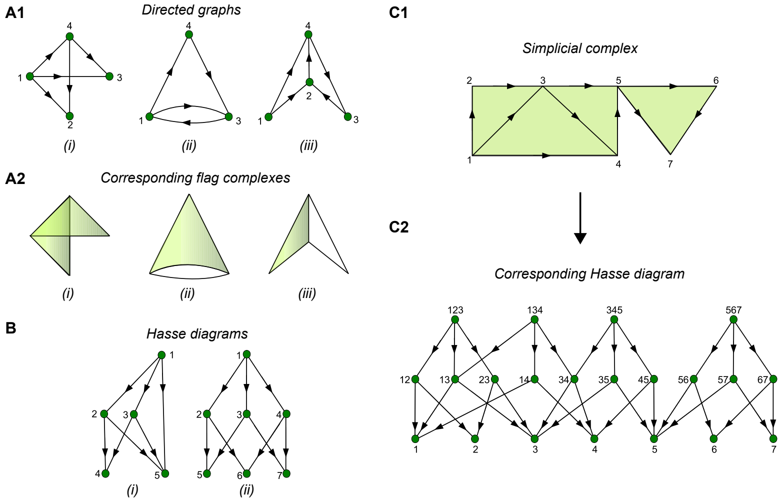

Figure S6: A1: Examples of directed graphs. (i) An acyclic graph. Vertex 1 is a source. Vertices 2 and 3 are sinks. (ii) A graph with one reciprocal connection, two cycles and no sources or sinks. (iii) A graph containing a cycle on the vertices 2, 3 and 4. Vertex 1 is a source. There are two directed paths from vertex 1 to vertex 3: 1-4-3 and 1-2-4-3. A2: Directed flag complexes associated to the directed graphs in A1. (i) Two 2-simplices correspond to the two directed 3-cliques. (ii) One 2-simplex corresponds to one directed 3-clique. The reciprocal connection contributes an additional 1-simplex, but not another 2-simplex. (iii) One 2-simplex corresponds to a single directed 3-clique. The cycle does not contribute a 2-simplex. B: (i) A Hasse diagram that is not stratified, due to the edge from the vertex 1 to 5. (ii) A stratified Hasse diagram, where vertices 5, 6, and 7 are the vertices of level 0, vertices 2, 3, and 4 are of level 1, and vertex 1 is of level 2. This is also an admissible Hasse diagram, where the outgoing edges are ordered from left to right. Vertex 2 is a front face of vertex 1, while vertex 3 is neither a front nor a back face of a vertex 1, and vertex 4 is back face of a vertex 1. C1: The geometric realization of a simplicial complex consisting of seven 0-simplices (labeled 1,...,7), ten 1-simplices, and four 2-simplices. The orientation on the edges is denoted by arrows, i.e., the tail of an arrow is its source vertex, while the head of an arrow is its target. C2: The Hasse diagram corresponding to the simplicial complex above. Level k vertices correspond to k-simplices of the complex and are labeled by the ordered sets of vertices that constitute the corresponding simplex. Note that, e.g., vertex 23 is a back face of a vertex 123 and a front face of a vertex 234.

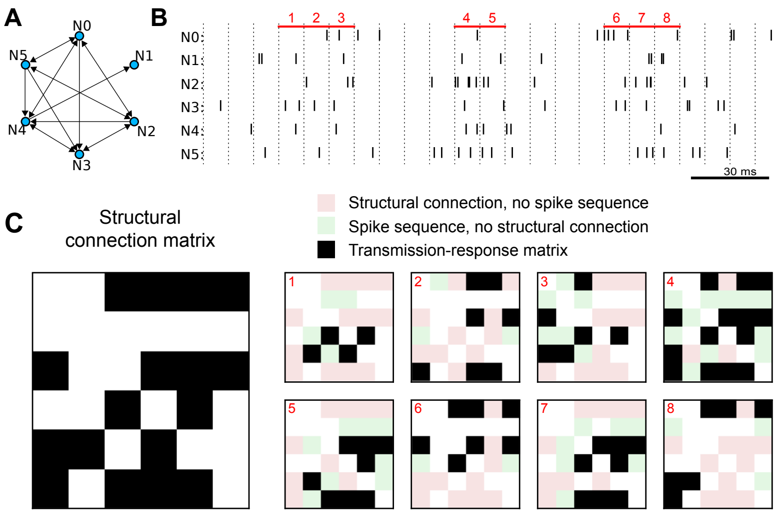

Figure S7: A: Exemplary connectivity motif of six neurons B: Random spikes generated for all six neurons. Dashed lines indicate time steps of 10 ms. C: Left: Structural connection matrix, Right: Resulting transmission-response (TR) matrices for eight time steps indicated in B. Additionally, structural connections that are not active in the TR graph are indicated in red, and instances of a spike sequence within $\Delta t = 7.5$ms that lack a structural connection are indicated in green.

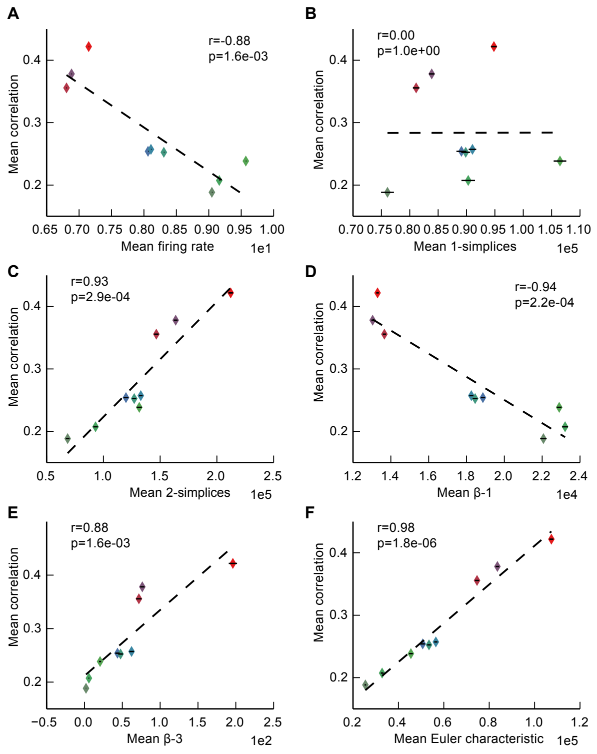

Figure S8: A: Mean pairwise correlation of neurons in microcircuit during the first 250ms of each stimulus, vs. mean firing rate in the same time range. B-F: Correlation vs. topological metrics.

2 SUPPLEMENTARY METHODS

2.1 Proof: Maximal Directionality of Directed Simplices

We defined the *directionality* of $\mathcal{G}$ to be the sum over all vertices of the square of their signed degrees, i.e.,

$$\mathrm{Dr}(\mathcal{G}) = \sum_{v\in V} \mathrm{sd}(v)^2.$$

Let $\mathcal{G}_n$ denote a directed n-simplex, i.e., a fully connected directed graph on $n+1$ vertices such that every fully connected subgraph has a unique source and a unique sink, and which therefore has no reciprocal connections.

PROPOSITION 1. *If $\mathcal{G}$ is a directed graph on $n+1$ vertices, then $\mathrm{Dr}(\mathcal{G}) \leq \mathrm{Dr}(\mathcal{G}_n)$. If additionally $\mathcal{G}$ is a fully connected directed graph without reciprocal connections, then equality holds if and only if $\mathcal{G}$ is isomorphic to $\mathcal{G}_n$ as a directed graph.*

PROOF. We prove this result by induction on n. Observe that it holds trivially for $n = 1$, since every complete directed graph on two vertices is a directed 1-simplex. Assume that the proposition holds for all $n < N$, and let $\mathcal{G}$ be a complete directed graph on $N + 1$ vertices, $v_0, ..., v_N$. Without loss of generality, suppose that the signed degree of v_0 is maximal, i.e., $\mathrm{sd}(v_0) \geq \mathrm{sd}(v_i)$ for all $i \geq 1$.

Create a new graph $\mathcal{G}'$ by reversing the direction of each edge that is directed away from v_0, so that v_0 becomes a sink in $\mathcal{G}'$. Let $v'_0, \ldots, v'_N$ denote the vertices of $\mathcal{G}'$, such that v'_i is the vertex corresponding to v_i. Then $\mathrm{sd}(v'_0) = N$, and for each $i \geq 1$ such that the edge from v_0 to v_i was reversed, one has

$$\mathrm{sd}(v'_i) = \mathrm{sd}(v_i) - 2,$$

since only edges involving v_0 can change when passing from $\mathcal{G}$ to $\mathcal{G}'$.

Let k be the number of edges in $\mathcal{G}$ that change orientation in passing from $\mathcal{G}$ to $\mathcal{G}'$. Observe that

$$\begin{aligned}
\mathrm{Dr}(\mathcal{G}') &= (\mathrm{sd}(v_0) + 2k)^2 + \sum_{\substack{\text{edge from } v_0\\ \text{unchanged}}} \mathrm{sd}(v_j)^2 + \sum_{\substack{\text{edge from } v_0\\ \text{reversed}}} (\mathrm{sd}(v_i) - 2)^2 \\
&= \mathrm{Dr}(\mathcal{G}) + 4 \sum_{\substack{\text{edge from } v_0\\ \text{reversed}}} \underbrace{(\mathrm{sd}(v_0) - \mathrm{sd}(v_i))}_{\geq 0\,\forall i} + 4k^2 + 4k \\
&\geq \mathrm{Dr}(\mathcal{G}),
\end{aligned}$$

where equality holds if and only if $k = 0$, i.e., if v_0 is already a sink in $\mathcal{G}$. Note that if $k \neq 0$, so that v_0 is not already a sink in $\mathcal{G}$, then no vertex of $\mathcal{G}$ is a sink, since $\mathrm{sd}(v_0) \geq \mathrm{sd}(v_i)$ for all $i \geq 1$, and a vertex v of a complete directed graph on $N + 1$ vertices is a sink if and only if $\mathrm{sd}(v) = N$.

The subgraph $\mathcal{G}''$ of $\mathcal{G}'$ spanned by the vertices $v'_1, \ldots, v'_N$ is a complete directed graph on N vertices. By induction, $\mathrm{Dr}(\mathcal{G}'') \leq \mathrm{Dr}(\mathcal{G}_{N-1})$, with equality holding if and only if $\mathcal{G}''$ is a directed $(N-1)$-simplex. Therefore

$$\mathrm{Dr}(\mathcal{G}) \leq \mathrm{Dr}(\mathcal{G}') = \mathrm{Dr}(\mathcal{G}'') + N^2 \leq \mathrm{Dr}(\mathcal{G}_{N-1}) + N^2 = \mathrm{Dr}(\mathcal{G}_N),$$

with equality holding only if v_0 is a sink in $\mathcal{G}$, and $\mathcal{G}''$ is a directed $(N-1)$-simplex, i.e., if and only if $\mathcal{G}$ is a directed N-simplex.

Directionality of directed simplices is proportional to the cube of their dimension.

LEMMA 2. *For each* $n \geq 1$,

$$\mathrm{Dr}(\mathcal{G}_n) = \frac{n(n+1)(n+2)}{3}.$$

PROOF. Let $v_0, \ldots v_n$ be the vertices of $\mathcal{G}_n$ ordered from sink to source. It is easy to see that the associated sequence of signed degrees takes the form

$$n, n-2, n-4, \ldots, 2, 0, -2, \ldots, 4-n, 2-n, -n,$$

if n is even, and

$$n, n-2, n-4, \ldots, 1, -1, \ldots, 4-n, 2-n, -n,$$

if n is odd. Hence

$$\mathrm{Dr}(\mathcal{G}_n) = \begin{cases} 2\left(\sum_{k=1}^{n} k^2 - \sum_{j=1}^{\frac{n}{2}} (2j-1)^2\right), & n \text{ even} \\ 2\left(\sum_{k=1}^{n} k^2 - \sum_{j=1}^{\frac{n-1}{2}} (2j)^2\right), & n \text{ odd} \end{cases}.$$

Recall the well known formulas

$$\sum_{k=1}^{n} k^2 = \frac{n(n+1)(2n+1)}{6}, \quad \text{and} \quad \sum_{k=1}^{n} k = \frac{n(n+1)}{2}.$$

If n is even then, using these formulas,

$$\sum_{j=1}^{\frac{n}{2}} (2j-1)^2 = 4\sum_{j=1}^{\frac{n}{2}} j^2 - 4\sum_{j=1}^{\frac{n}{2}} j + \frac{n}{2} = \frac{n(n+1)(n+2)}{6} - \frac{n(n+2)}{2} + \frac{n}{2} = \frac{n(n-1)(n+1)}{6}.$$

Similarly for n odd,

$$\sum_{j=1}^{\frac{n-1}{2}} (2j)^2 = 4\sum_{j=1}^{\frac{n-1}{2}} j^2 = \frac{n(n-1)(n+1)}{6}.$$

Hence,

$$\mathrm{Dr}(\mathcal{G}_n) = 2\left(\frac{n(n+1)(2n+1)}{6} - \frac{n(n-1)(n+1)}{6}\right) = \frac{n(n+1)(n+2)}{3},$$

as claimed.

2.2 Generation of Directed Flag Complexes

2.2.1 Hasse Diagrams

A *Hasse diagram*, otherwise known as a directed acyclic graph, is a directed graph $\mathcal{H} = (V, E, \tau)$ with no oriented cycles (Figure S6B). Hasse diagrams can be used to encode various combinatorial, geometric, and topological structures, such as posets, cubical complexes and simplicial complexes. Below we explain in detail how to encode simplicial complexes via Hasse diagrams.

A Hasse diagram $\mathcal{H}$ is said to be *stratified* if for each $v \in V$, every path from v to any sink in $\mathcal{H}$ has the same length. Thus in a stratified Hasse diagram the vertices are naturally partitioned into disjoint strata,

where every directed path from a vertex in the k-th stratum V_k to any sink is of length k. In particular, the 0-th stratrum V_0 is the set of sinks of $\mathcal{H}$. Moreover, for all $e \in E$, there exists $k > 0$ such that $\tau_1(e) \in V_k$ and $\tau_2(e) \in V_{k-1}$.

A Hasse diagram $\mathcal{H}$ is said to be oriented if for every vertex $v \in \mathcal{H}$, it is equipped with a linear ordering of the set E_v of edges with source v. A Hasse diagram is said to be *admissible* if it is stratified and oriented.

Vertices in the k-th stratum of a stratified Hasse diagram $\mathcal{H}$ are said to be *of level k*. If $k < n$, and v, u are vertices of levels k and n respectively, then we say that v is a *face* of u if there is a directed path in $\mathcal{H}$ from u to v. If $\mathcal{H}$ is also oriented and therefore admissible, and there is a directed path $(e_1, ..., e_{n-k})$ from u to v such that e_i is the *first* element of $E_{\tau_1(e_i)}$ with respect to the ordering on that set, for all $1 \leq i \leq n-k$, we say that v is a *front face* of u. Similarly, v is a *back face* of u if there is a path $(e_1, ..., e_{n-k})$ from u to v such that e_i is the *last* element of the set $E_{\tau_1(e_i)}$ with resect to the ordering on it, for all $1 \leq i \leq n-k$. (Figure S6B(ii) and C2).

If $\mathcal{G} = (V, E, \tau)$ is a directed graph, then $\mathcal{G}$ can be equivalently represented by an admissible Hasse diagram with level 0 vertices V, level 1 vertices E, and directed edges from each $e \in E$ to its source and target. The ordering on the edges in the Hasse diagram is determined by the orientation of each edge e in $\mathcal{G}$.

Every directed simplicial complex $\mathcal{S}$ gives rise to an admissible Hasse diagram $\mathcal{H}_\mathcal{S}$ as follows (Figure S6C1 and C2). The level d vertices of $\mathcal{H}_\mathcal{S}$ are the d-simplices of $\mathcal{S}$. There is a directed edge from each d-simplex to each of its $(d-1)$-faces. The stratification on $\mathcal{H}_\mathcal{S}$ is thus given by dimension, and the orientation is given by the natural ordering of the faces of a simplex from front to back.

2.2.2 A Data Structure for Hasse Diagrams

The algorithm we use in order to produce the directed flag complex of a directed graph uses Hasse diagrams as both input and output. Hence the input directed graph $\mathcal{G} = (V, E, \tau)$ must first be turned into a Hasse diagram. The output of the algorithm is again an admissible Hasse diagram that encodes the directed flag complex of the graph.

We represent an admissible Hasse diagram $\mathcal{H}$ using vectors to store the references to the vertices of the diagram. Thus, each vertex $v \in \mathcal{H}$ stores the following information.

1. $\mathrm{Ver}(v)$: A vector of the level 0 vertices of $\mathcal{H}$ that is a list of the 0-faces of v ordered according to the orientation of $\mathcal{H}$. If v is at level 0, then $\mathrm{Ver}(v) = \emptyset$.
2. $\mathrm{Tar}(v)$: A vector of references to the vertices that are targets of edges with source v. If v is at level 0, then $\mathrm{Tar}(v) = \emptyset$.
3. $\mathrm{Src}(v)$: A vector of references to the vertices that are sources of edges with target v.

If $\mathcal{H}$ encodes a directed simplicial complex, then $\mathrm{Ver}(v)$ determines the simplex to which v corresponds, and the vectors $\mathrm{Tar}(v)$ and $\mathrm{Src}(v)$ determine the faces and co-faces of the simplex to which v corresponds, respectively.

If $\mathcal{H}$ is admissible Hasse diagram of maximal level d, then $\mathcal{H}$ is represented by an ordered set of d vectors, where for each $1 \leq n \leq d$, the n-th vector contains the references to all level n vertices.

In addition, for every vertex v in $\mathcal{H}$ of level $n \geq 1$ such that $\mathrm{Ver}(v) = [v_0, \ldots, v_i]$, the algorithm temporarily records an auxiliary vector U_v of references to level 0 vertices u in $\mathcal{H}$ that satisfy the following properties:

1. $u \neq v_i$ for all $0 \leq i \leq n$, and

2. for every $u \in U_v$ and every $0 \leq i \leq n$, there exists an edge in $\mathcal{G}$ from v_i to u.

The vector U_v is dismissed once its function has been fulfilled.

Let S_{int} denote the size of integer data types, and for any finite set X, let $|X|$ denote its cardinality. Each edge of an Hasse diagram $\mathcal{H}$ is stored in two vertices of the diagram. If each reference requires S_{int} storage, then we require $O(|E| \cdot S_{\text{int}})$ space to store all references, where E is the edge set of $\mathcal{H}$. In addition, each vertex v in $\mathcal{H}$ the corresponding data structure stores the vectors $\text{Ver}(v)$, $\text{Tar}(v)$ and $\text{Src}(v)$, as explained above, which requires an additional $O(S_{int} \cdot d)$ of space per vertex. The total size of a Hasse diagram is thus bounded by $O((S_{\text{int}} \cdot d) \cdot |V| + |E| \cdot S_{\text{int}})$, where V is the vertex set of $\mathcal{H}$. In particular, the required storage space grows linearly with the number of vertices and with the number of edges.

Algorithm 1 : Directed flag complex generation.

Input: A Hasse diagram $\mathcal{H}_{in}$ encoding the directed graph $\mathcal{G} = (V, E, \tau)$, (cf. ST4.1.1).
Output: A Hasse diagram $\mathcal{H}$ representing the directed flag complex associated to $\mathcal{G}$.

1: Set $\mathcal{H} = \mathcal{H}_{in}$
2: **for** every level 1 vertex $e \in \mathcal{H}$ **do**
3:     **if** exist e_1, e_2 such that $\tau_1(e_1) = \tau_1(e)$, $\tau_1(e_2) = \tau_2(e)$ and $\tau_2(e_1) = \tau_2(e_2) = u$ **then**
4:         Add u to U_e;
5:     **end if**
6: **end for**
7: $dim = 2$;
8: **repeat**
9:     *next_level_nodes* – empty vector of references to nodes;
10:     **for** top–level vertex $e \in \mathcal{H}$ **do**
11:         **for** Every $u \in U_e$ **do**
12:             Create a node t_u of a Hasse diagram;
13:             $\text{Ver}(t_u) = [\text{Ver}(e), u]$;
14:             $U_{t_u} = U_e$;
15:             Add e to $\text{Tar}(t_u)$;
16:             Add t_u to $\text{Src}(e)$;
17:             **for** Every $bd \in \text{Tar}(e)$ **do**
18:                 **for** Every $cbd \in \text{Src}(bd)$ **do**
19:                     **if** u is the last vertex in $\text{Ver}(cbd)$ **then**
20:                         Add cbd to $\text{Tar}(t)$;
21:                         Add t_u to $\text{Src}(cbd)$;
22:                         $U_{t_u} = U_{t_u} \cap U_{cbd}$;
23:                     **end if**
24:                 **end for**
25:             **end for**
26:             Add t_u to *next_level_nodes*;
27:         **end for**
28:     **end for**
29:     Add *next_level_nodes* to $\mathcal{H}$;
30:     $dim = dim + 1$;
31: **until** *next_level_nodes* = $\emptyset$
32: Return $\mathcal{H}$;

2.2.3 Directed Flag Complex Generation Algorithm

The discussion below refers to the pseudo-code given in Algorithm 1.

Line 1: The Hasse diagram $\mathcal{H}$ takes as an initial value the input Hasse diagram encoding a directed graph $\mathcal{G} = (V, E, \tau)$.

Lines 2 - 7: The *for* loop initialises the creation of the vectors U_e for level 1 vertices $e \in \mathcal{H}$. For every level 1 vertex e, the vector U_e stores the references to all the level 0-vertices that, together with e, will form a level 2 vertex.

The *if* condition (Line 3) ensures that whenever the code finds two level 1 vertices $\{e_1, e_2\}$ and a level 0 vertex u that satisfy the conditions, the vertex u will be the terminal vertex of the level 2 vertex t_u that will be created in the first iteration of the *repeat-until* loop (Line 8). Notice also that the same *if* condition ensures that each triple of level 1 vertices (e, e_1, e_2) is naturally ordered as the front, middle and back faces of a an oriented 2-simplex associated with t_u. In particular, e is the front face of t_u, and hence the ordering of $\mathrm{Ver}(e)$ can be extended to ordering of $\mathrm{Ver}(t_u)$, as in Line 13.

Lines 8 - 31: This *repeat-until* loop is where the complex is generated, where each iteration increases the dimension by 1.

Fix $n \geq 1$, and suppose by induction that all vertices of level less than or equal to n have been constructed. Fix a level n vertex s with $\mathrm{Ver}(s) = [v_0, \ldots, v_n]$ and let $u \in U_s$. By definition of the set U_s, the code creates a vertex t_u (Line 10), creates $\mathrm{Ver}(t_u)$ as $[\mathrm{Ver}(s), u] = [v_0, \ldots, v_n, u]$, so u is the last vertex in $\mathrm{Ver}(t_u)$ (Line 11), and initiates U_{t_u} as U_s (Line 12). In the next two lines (Lines 13 and 14) s becomes a target of t_u and t_u a source of s. Next, in the two *for* loops of Lines 15 and 16, the code checks for each level $n-1$ vertex bd that is a target of s, and every level n vertex cbd that is a source of bd, whether u is the last vertex in $\mathrm{Ver}(cbd)$ (Line 17). In that case cbd becomes a target of t_u and t_u becomes a source of cbd (Lines 18, 19). Since u is the *last* vertex in $\mathrm{Ver}(t_u)$, it must be the last vertex in any face of t_u that contains it for the orientation to be preserved, whence the restriction in Line 17. This accounts of all the co-dimension 1 faces (targets) of t_u that are different from s, and by induction hypothesis these faces are already constructed. Hence t_u is declared a new level $n+1$ vertex in $\mathcal{H}$. Since in a directed simplicial complex (in particular a directed flag complex) every simplex is characterised by its ordered list of vertices, a level $n+1$ vertex t in $\mathcal{H}$ with $\mathrm{Ver}(t) = [v_0, \ldots, v_n, u]$ can only be constructed once, and hence is equal to t_u. On the other hand, Line 20 ensures that all potential vertices of level larger than $n+1$ of which t_u is a target will be accounted for. It follows that Algorithm 1 does indeed construct construct the Hasse diagram corresponding to the directed flag complex of the input graph.

If w is a level $n+1$ vertex with $\mathrm{Ver}(w) = [v_0, \ldots v_n, u]$, then the level n vertex v that is the front face of w is a target of w, and u is clearly not present in $\mathrm{Ver}(v)$. On the other hand, u is listed in U_v. From Lines 12 and 20 of the algorithm it is clear that $U_w \subset U_v$ and moreover that $u \notin U_w$, and so the inclusion is proper. The cardinalities of the sets $U_{(-)}$ are therefore strictly decreasing for the newly created vertices. New level $n+1$ vertices are created only if there exists at least one level n vertex t, such that $U_t \neq \emptyset$. Since the cardinality of the $U_{(-)}$ decreases with each iteration of the *repeat-until* loop (Line 6), the algorithm will terminate.

2.3 In Vitro Slice Experiments

2.3.1 Slice Preparation

Experiments were carried out according to the Swiss national and institutional guidelines. Fourteen- to sixteen-day-old nonanesthetized Wistar rats were quickly decapitated, and their brains were carefully removed and placed in iced artificial cerebrospinal fluid (ACSF). Slices (300 μm) were cut on an HR2 vibratome (Sigmann Elektronik). Parasagittal slices, $1.7 - 2.2\, mm$ lateral to the midline, were cut to access primary somatosensory cortex (SSC; above the anterior extremity of the hippocampus ±1 mm). Slices were incubated at 37°C for 30 to 60 min and then left at room temperature until recording. Cells were visualized

by infrared differential interference contrast video microscopy using a VX55 camera (Till P-hotonics) mounted on an upright BX51WI microscope (Olympus). Layer 5 thick-tufted pyramidal cells (L5TTPCs) were selected according to their large soma size ($15 - 25\mu m$) and their apparent large trunk of the apical dendrite. Care was taken to use only parallel slices (i.e., slices that had a cutting plane parallel to the course of the apical dendrites and the primary axonal trunk). This ensured sufficient preservation of the PCsO axonal and dendritic arborizations.

2.3.2 Chemicals and Solutions

Slices were continuously superfused with ACSF containing 125 mM NaCl, 25 mM NaHCO3, 2.5 mM KCl, 1.25 mM NaH2PO4, 2 mM CaCl2, 1 mM MgCl2, and 25 mM D-glucose bubbled with 95% O2 and 5% CO2. The intracellular pipette solution contained 110 mM potassium gluconate, 10 mM KCl, 4 mM ATP-Mg, 10 mM phosphocreatine, 0.3 mM GTP, 10 Hepes, and 13 mM biocytin adjusted to pH 7.3-7.4 with 5 M KOH. Osmolarity was adjusted to 290-300 mOsm L_{-1} with D-mannitol ($25 - 35$ mM). The membrane potential values given were not corrected for the liquid junction potential, which is approximately -14 mV. Chemicals were from Sigma Aldrich or Merck.

2.3.3 Electrophysiological Recordings

Multiple somatic whole-cell recordings (6 to 12 cells simultaneously) were performed with Multiclamp 700B amplifiers (Molecular Devices) in the current clamp mode at 34 ± 1°C bath temperature. Data acquisition was performed through an ITC-1600 board (Instrutech) connected to a PC running a custom-written routine (PulseQ) under IGOR Pro (Wavemetrics). Sampling rates were 5 kHz, and the voltage signal was filtered with a 2-kHz Bessel filter. Patch pipettes were pulled with a Flaming/Brown micropipette puller P-97 (Sutter Instruments) or a DMZ puller (Zeitz Instruments) and had an initial resistance of 3-8MΩ. Recordings were achieved with custom C++ software that controlled manipulators, amplifiers, oscilloscopes, pipette pressure, and video display.

2.3.4 Stimulation Protocols

Monosynaptic, direct excitatory connections were identified by stimulation of a presynaptic cell with a $20 - 70$ Hz train of $5 - 15$ strong and brief current pulses ($1 - 2$ nA, $2 - 4$ ms) followed by a so-called recovery test response $0.5s$ after the end of the train (not shown in the traces), all precisely and reliably eliciting action potentials (APs).

2.3.5 Final Somatic Positions

The soma positions were recorded relative to an arbitrary reference point, and the z-axis was oriented perpendicular to the surface of the slice. After morphological stainings were ready, the y-axis axis was rotated around the z-axis axis to match the orientation of the apical dendrites. The x-axis was rotated by the same amount and remained orthogonal to the other two axes.

2.3.6 Connection Amplitudes

The amplitude of excitatory postsynaptic potentials (EPSPs) was measured for events that followed a resting period of at least 15 s, during which time the presynaptic neurons were not stimulated to produce APs.

2.4 Optimization of the Parameters for the Transmission-Response Matrices

Starting with firing data from spontaneous activity in the reconstructed microcircuit, we generated sequences of 20 transmission-response matrices for $\Delta t_i \in \{1, 2, 5, 10, 20, 50, 100\}$ ms, thus creating 49

such sequences corresponding to every possible choice of the pair $(\Delta t_1, \Delta t_2)$. We refer to each of these sequences as the *true transmission-response matrices* corresponding to the pair $(\Delta t_1, \Delta t_2)$. Here, we describe the procedure for optimizing the choice of the time intervals Δt_1 and Δt_2 so that the associated true transmission-response matrices best reflect the actual successful transmission of signals between neurons in the microcircuit.

2.4.1 Properties of the Transmission-Response Matrix

The nonzero coefficients in a transmission-response matrix are a subset of those in the structural matrix. Due to the partly stochastic behavior of the *in silico* microcircuit, the subset will vary even for subsequent applications of the same stimulus. In fact, even an exact repetition of the same conditions will lead to different transmission-response matrices, if the random number generator is seeded differently. It follows that the generation of the transmission-response matrices for a given stimulus should be considered as a stochastic process. With the correct choice of the parameters Δt_i, the matrices should reflect how the microcircuit processes a stimulus and thus take into account parameters of neural processing, such as pre-post synaptic interaction.

To find parameters Δt_1 and Δt_2 that maximize the degree to which neural processing is captured by the transmission-response matrices, we first develop a stochastic model for synaptic firing that takes into account neural processing and that depends on Δt_1 and Δt_2. For the purpose of this analysis, we assume that the true transmission-response matrices are compatible with this model.

Based upon our model for synaptic firing, we formulate a simplified model that ignores neural processing. For this simplified model and for any choice of parameters Δt_1 and Δt_2, we explain how to obtain transmission-response matrices from actual firing data, by shuffling the firing data appropriately, then applying the algorithm for generating a transmission-response matrix of the previous section. Finally, for each choice of the parameters Δt_1 and Δt_2, we compare the true transmission-response matrices for spontaneous activity in the reconstructed microcircuit to those obtained by the simplified generation process. The parameters that we work with in the main body of the paper are the Δt_1 and Δt_2 that maximize the difference (measured by the ratio of the numbers of ones in the matrices) between the actual transmission-response matrices and those resulting from the simplified model.

2.4.2 Stochastic Model with Neural Processing

Fix time intervals Δt_1 and Δt_2. Let $A = (a_{ij})$ denote the structural matrix of a reconstructed microcircuit, and let $A(n) = (a^n_{ij})$ denote the transmission-response matrix of the n-th time bin, based on firing data from a trial of simulated activity in the microcircuit, for the given intervals Δt_1 and Δt_2. By Condition (1) above, if $a^n_{ij} = 1$ for any n, then $a_{ij} = 1$. It is reasonable to consider A to be static, at least over the time periods considered here.

We want to compute the probability that $a^n_{ij} = 1$, given that $a_{ij} = 1$, so we need to determine on which parameters and properties this probability depends. According to the definition of transmission-response matrices, a presynaptic and a postsynaptic spike are required for a^n_{ij} to be 1. To simplify the analysis somewhat, we assume that each neuron n_i has a *time-dependent, instantaneous firing rate* $F^i(t)$ that determines spiking probability at time t, i.e., spiking can be described as an inhomogeneous Poisson process. Under this assumption, the expected number $m^i_{\Delta t_1}(t_0)$ of spikes of neuron n_i in the interval $[t_0, t_0 + \Delta t_1]$ can be computed as

$$m^i_{\Delta t_1}(t_0) = \int_{t_0}^{t_0+\Delta t_1} F^i(u)du.$$

If $K^i_{\Delta t_1}(t_0)$ denotes the probability that neuron n_i spikes at least once in the interval $[t_0, t_0 + \Delta t_1]$, then

$$K^i_{\Delta t_1}(t_0) = 1 - \mathcal{P}\big(m^i_{\Delta t_1}(t_0)\big) = 1 - e^{-m^i_{\Delta t_1}(t_0)},$$

where $\mathcal{P}(\lambda)$ is the Poisson probability mass function with parameter λ at 0. (Recall that if X is a random variable that counts the number of spikes of neuron n_i in the interval $[t_0, t_0 + \Delta t_1]$, then $\mathcal{P}\big(m^i_{\Delta t_1}(t_0)\big)$ is the probability that $X = 0$.) If the change in $F^i(t)$ is slow compared to Δt_1, then $m^i_{\Delta t_1}(t) \approx F^i(t) \cdot \Delta t_1$. Moreover, $1 - \mathcal{P}(\lambda) \approx \lambda$ for small values of λ. For small enough Δt_1, the expected number $m^i_{\Delta t_1}(t_0)$ of spikes of neuron n_i will certainly be small, and change in $F^i(t)$ will be slow in compared to Δt_1, so that we may assume that

$$K^i_{\Delta t_1}(t_0) \approx F^i(t_0) \cdot \Delta t_1.$$

For the postsynaptic spike the situation is more complicated. As there is a causal relation between presynaptic and postsynaptic firing, mediated by synaptic transmission, we need to estimate the conditional probability of at least one postsynaptic spike, given that at least one presynaptic spike occured. Let n_i and n_j denote neurons such that $a_{ij} = 1$. Let $s_0 \in [t_0, t_0 + \Delta t_1]$ denote the time of the first presynaptic spike in this interval. Let $X^j_{\Delta t_2}(s_0)$ denote the random variable whose value is the number of times neuron n_j spiked in the time window $[s_0, s_0 + \Delta t_2]$. Let $Y^i_{\Delta t_1}(t_0)$ denote the random variable whose value is the number of times neuron n_i spiked in the time interval $[t_0, t_0 + \Delta t_1]$. We need to estimate the conditional probability

$$P\big(X^j_{\Delta t_2}(s_0) > 0 \,|\, Y^i_{\Delta t_1}(t_0) > 0\big).$$

The nature of this interaction is very intricate and depends on the identities of the presynaptic and postsynaptic neurons, the spiking history of the presynaptic neuron before s_0, and all other synaptic input the postsynaptic neuron received. It can be described as governed by some function G^{ij} modulating the spiking probability of the postsynaptic neuron n_j. This function takes as parameters the expected number of spikes of neuron n_j in the interval $[s_0, s_0 + \Delta t_2]$, the time t_0, and the "spiking history" of the presynaptic neuron n_i until s_0, which we write as a function $s^i_*(t)$ evaluated at s_0, giving rise to the expression

$$P\big(X^j_{\Delta t_2}(s_0) > 0 \,|\, Y^i_{\Delta t_1}(t_0) > 0\big) = 1 - e^{-G^{ij}(m^j_{\Delta t_2}(s_0), t_0, s^i_*(s_0))}.$$

Summarizing the analysis above, the following formula provides a good estimate of the probability that $a^n_{ij} = 1$ if $a_{ij} = 1$, for small enough Δt_1 and Δt_2, where s_0 denotes the time of the first presynaptic spike in the interval $[n\Delta t_1, (n+1)\Delta t_1]$ and $t_0 = n\Delta t_1$.

$$\begin{aligned} P\left(a^n_{ij} = 1 | a_{ij} = 1\right) &= \left(1 - e^{-m^i_{\Delta t_1}(t_0)}\right) \cdot \left(1 - e^{-G^{ij}(m^j_{\Delta t_2}(s_0), t_0, s^i_*(s_0))}\right) \\ &\approx F^i(t_0) \cdot \Delta t_1 \cdot G^{ij}\big(F^j(s_0) \cdot \Delta t_2, t_0, s^i_*(s_0)\big). \end{aligned} \tag{S1}$$

This conditional probability encodes not only the distinctive features of the structural connectivity (via a_{ij}) but also the potentially stimulus-dependant neuron-specific firing rates (via F^i and F^j) and their co-variation. Most crucially, it captures the stimulus-dependent functional modulation of postsynaptic firing by a presynaptic spike as well. We assume that the true transmission-reponse matrices capture the actual transmission of spikes according to the model of synaptic firing described by this formula.

2.4.3 Null Hypotheses: No Neural Processing

We introduce here a simplified model of synaptic spiking that is based upon formula [S1] but that ignores pre-post synaptic interaction. We then explain how to obtain transmission-response matrices that correspond to this simplified model from firing data arising from simulated activity.

We begin by setting each G^{ij} to be the projection onto the first component, ignoring the pre-post synaptic interaction. After this simplification, the approximation obtained in the previous section now reads

$$P(a_{ij}^n = 1 | a_{ij} = 1) \approx F^i(t_0) \cdot F^j(s_0) \cdot \Delta t_1 \cdot \Delta t_2,$$

where s_0 denotes the time of the first presynaptic spike that occurs in the interval $[n\Delta t_1, (n+1)\Delta t_1]$ and $t_0 = n\Delta t_1$, as before. Since this drastic simplification neglects the central aspect of neural computation - pre-post synaptic interaction - it gives rise to control cases for each pair of parameters $(\Delta t_1, \Delta t_2)$ and each choice of firing rate functions $F^i(t)$. Comparison of the true transmission-response matrices for each pair of parameters to the corresponding control matrices for the same pair and a specific choice of the functions $F^i(t)$ will allow us to determine values for Δt_1 and Δt_2 for which the true transmission-response matrix optimally reflects neural processing.

We assume moreover that the individual firing rates consist of a neuron-dependent frequency that is up- or down-regulated by a global time series, i.e., that $F^i(t) = f(i) \cdot F(t)$, for some function $F(t)$ and some constant $f(i)$ for each neuron n_i. Transmission-response matrices corresponding to this simplified model for fixed Δt_1 and Δt_2, which we call *simplified transmission-response matrices*, can be generated by first shuffling all recorded spikes from simulated activity in the reconstructed microcircuit, while preserving both the number of spikes per neuron and per time bin, then applying the usual transmission-response matrix generation method.

2.4.4 Optimization of Parameters

The difference between the true transmission-response matrices and the control case described above is a consequence of the pre-post synaptic interaction. Comparison with the control case enables us therefore to measure how well that interaction is captured in the true transmission-response matrices. In particular, it is reasonable to optimize the parameters Δt_1 and Δt_2 so that the difference between the true transmission response matrices arising from actual simulation data and those arising in the control cases is maximized, as a maximal difference indicates that the effect of the pre-post synaptic interaction is captured optimally by the true transmission-response matrices.

The comparison between the true transmission-response matrices and the control cases was carried out by first producing 20 true transmission-response matrices and 20 simplified transmission-response matrices based on firing data obtained from spontaneous activity in the reconstructed microcircuit for every pair $(\Delta t_1, \Delta t_2)$, where $\Delta t_i \in \{1, 2, 5, 10, 20, 50, 100\}$ ms for $i = 1, 2$. The number of ones in each matrix was then computed and the average taken over each set of 20 matrices. Since no stimulus was applied to the microcircuit, the averages computed are meaningful, since the firing data should be fairly homogeneous across the time bins.

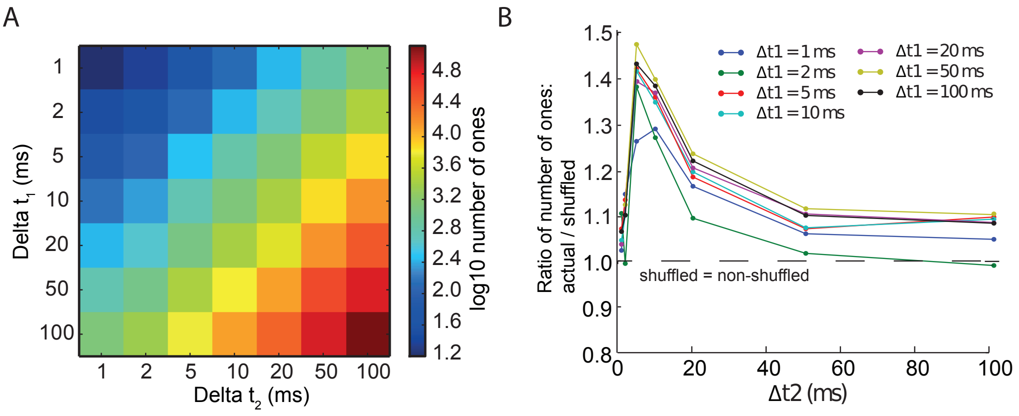

The average number of ones in the transmission-response matrix arising from simulated actitivity in the reconstructed microciruit, as a function of Δt_1 and Δt_2, is illustrated in the figure above, which shows the ratio of the average number of ones in the true transmission-response matrices to the average number of ones in the simplified transmission-response matrices, for various values of Δt_1 and Δt_2. In all cases we find that the maximum lies between $\Delta t_2 = 5$ ms and $\Delta t_2 = 10$ ms, leading us to choose to work with $\Delta t_2 = 10$ ms. For Δt_1 we find a maximum at 50 ms, but we use $\Delta t_1 = 5$ ms (for which the maximum ratio is only slightly lower than for $\Delta t_1 = 50$ ms) instead to avoid more than one spike per neuron per bin.
